# Effectiveness and implementation of lower-intensity weight management interventions delivered by the non-specialist workforce in postnatal women: a mixed-methods systematic review

**DOI:** 10.3389/fpubh.2024.1359680

**Published:** 2024-03-28

**Authors:** Mackenzie Fong, Ryan Patrick William Kenny, Katie Thomson, Amrita Jesurasa, Amber Lavans, Maddey Patterson, Letitia Sermin-Reed, Giang Nguyen, Maria Raisa Jessica Aquino, Emer Cullen, Hannah O'Keefe, Malcolm Moffat, Nicola Heslehurst

**Affiliations:** ^1^Population Health Sciences Institute, Faculty of Medical Sciences, Newcastle University, Newcastle-upon-Tyne, United Kingdom; ^2^NIHR Applied Research Collaboration (ARC) North East and North Cumbria, Newcastle-upon-Tyne, United Kingdom; ^3^Fuse, The Centre for Translational Research in Public Health, Newcastle-upon-Tyne, United Kingdom; ^4^NIHR Innovation Observatory, Newcastle University, Newcastle-upon-Tyne, United Kingdom; ^5^Evidence Synthesis Group, Newcastle University, Newcastle-upon-Tyne, United Kingdom; ^6^Primary Care Division, Public Health Wales, Cardiff, United Kingdom

**Keywords:** postnatal, weight management, obesity, low intensity, primary care, community care

## Abstract

Lower-intensity interventions delivered in primary and community care contacts could provide more equitable and scalable weight management support for postnatal women. This mixed-methods systematic review aimed to explore the effectiveness, implementation, and experiences of lower-intensity weight management support delivered by the non-specialist workforce. We included quantitative and qualitative studies of any design that evaluated a lower-intensity weight management intervention delivered by non-specialist workforce in women up to 5 years post-natal, and where intervention effectiveness (weight-related and/or behavioural outcomes), implementation and/or acceptability were reported. PRISMA guidelines were followed, and the review was prospectively registered on PROSPERO (CRD42022371828). Nine electronic databases were searched to identify literature published between database inception to January 2023. This was supplemented with grey literature searches and citation chaining for all included studies and related reviews (completed June 2023). Screening, data extraction and risk of bias assessments were performed in duplicate. Risk of bias was assessed using the Joanna Briggs Institute appraisal tools. Narrative methods were used to synthesise outcomes. Seven unique studies described in 11 reports were included from the Netherlands (*n* = 2), and the United Kingdom, Germany, Taiwan, Finland, and the United States (*n* = 1 each). All studies reported weight-related outcomes; four reported diet; four reported physical activity; four reported intervention implementation and process outcomes; and two reported intervention acceptability and experiences. The longest follow-up was 13-months postnatal. Interventions had mixed effects on weight-related outcomes: three studies reported greater weight reduction and/or lower postnatal weight retention in the intervention group, whereas four found no difference or mixed effects. Most studies reporting physical activity or diet outcomes showed no intervention effect, or mixed effects. Interventions were generally perceived as acceptable by women and care providers, although providers had concerns about translation into routine practice. The main limitations of the review were the limited volume of evidence available, and significant heterogeneity in interventions and outcome reporting which limited meaningful comparisons across studies. There is a need for more intervention studies, including process evaluations, with longer follow-up in the postnatal period to understand the role of primary and community care in supporting women’s weight management. Public Health Wales was the primary funder of this review.

## 1 Introduction

Pregnancy and the postnatal period are life course stages of steep weight gain for many women (1). While is it normal and healthy for women to gain weight during pregnancy (i.e., through increases in fat mass and fluids and the weight of the foetus and placenta), many women gain more weight than is recommended. United States data show that almost half of pregnant women gain weight above National Academy of Medicine recommendations (previously Institute of Medicine) (2). For many women, losing the weight gained during pregnancy is challenging due to childcaring responsibilities, lack of time, domestic disruption, unpredictable schedules, impacts of pregnancy and delivery on the ability to exercise, and demands on emotional, cognitive, and material resources (3). On average, women gain approximately 14–15 kg during pregnancy, and at 1 year after delivery, 5–9 kg is retained (4, 5). Postpartum weight retention (PPWR; retaining weight gained during pregnancy) is a significant contributor to population-level overweight and obesity in women and, therefore, an issue of public health importance.

The postnatal period is an opportunity to intervene for longer-term health and well-being of women, and has potential to have beneficial inter-generational impacts on their children (6). Published systematic reviews report that high-intensity, structured, postnatal weight management interventions are beneficial for maternal weight loss, particularly when they combine diet and physical activity components and target women living with overweight and obesity (7–9). The evidence base also supports the need for flexible delivery of lower-intensity interventions (e.g., less frequent sessions, shorter intervention sessions), which may be more effective than high-intensity structured interventions in this population group (6). Embedding lower-intensity weight management interventions into existing primary care and community services that do not need to be delivered by staff with specialist nutrition/dietetic qualifications could be a way to provide more flexible and scalable weight management support. In the United Kingdom, some primary and community care contacts for young families are statutory and free at the point of care (e.g., child health visiting services). Therefore, this model of care whereby support is routinely offered to women without them needing to proactively seek it may be more equitable and a way to address socioeconomic disparities in maternal obesity (6). Embedding these interventions into primary and community care contacts may also enable support to be delivered over a longer period of time; some early childhood services in the United Kingdom (e.g., child health visiting services) are delivered until the child is school-aged, i.e., around 5 years postnatal. Further, as many women start to prepare for their next pregnancy in the years following their former pregnancy (3), postnatal weight management interventions can also serve as a preconception intervention for women who have subsequent pregnancies.

Existing systematic reviews to date have not focused on lower-intensity interventions that could be feasibly delivered in primary care and community settings by non-specialist staff and have tended to focus on the immediate postnatal period (e.g., 12-months) rather than over the longer-term. Additionally, systematic reviews to date have focused on intervention effectiveness from randomised controlled trials (RCTs) rather than integrating different study designs exploring postnatal weight management interventions more holistically. Therefore, this systematic review aimed to synthesise quantitative and qualitative evidence to explore lower-intensity interventions delivered by non-specialist workforce for weight management up to 5 years after pregnancy. This included assessing effectiveness on weight-, diet- and physical activity-related outcomes, factors influencing the implementation of these interventions, and the experiences of intervention deliverers and participants. The findings of this review can be used to inform the implementation of lower-intensity weight management interventions for postnatal women delivered in routine primary and community care contacts.

## 2 Methods

This systematic review was prospectively registered with PROSPERO (CRD42022371828) (available at https://www.crd.york.ac.uk/prospero/) and is reported as per PRISMA guidelines (10).

### 2.1 Search strategy and data sources

Nine electronic databases were searched from inception to 9th January 2023: MEDLINE, Embase, CINAHL, PsycINFO, Scopus, the Cochrane Central Register of Controlled Trials (CENTRAL), ProQuest Dissertation and Thesis, MIDIRS and CT.gov (Supplementary file S1). The search strategy was developed using keywords and MeSH headings by an information specialist (author, HOK). Grey literature searches of 38 websites were performed to supplement the bibliographic database searches (completed 10th February 2023, Supplementary file S2). Supplementary searches also included forwards and backwards citation chaining for all included studies and related systematic reviews (completed June 2023).

### 2.2 Inclusion and exclusion criteria

The inclusion and exclusion criteria were developed using PICOS criteria for population, intervention, comparison group, outcomes, and study design (Table 1). As we were interested in interventions that could be feasibly embedded into primary and community care, we included interventions that were lower-intensity, i.e., ≤1 intervention session/month based on a previous review of obesity management in primary care (11) that used the US Preventive Services Task Force (USPSTF) standards for intervention intensity. We included interventions that were not *required* to be delivered by staff with specialist nutrition/dietetic or weight management qualifications. Where the intervention was delivered by staff with these qualifications (e.g., dietitian led), authors reviewed the studies to determine whether the intervention was contingent on specialist skills and knowledge or if it would be transferrable to primary care or community contexts to be delivered by non-specialist staff, albeit with some level of training and skills development. For example, if in the research context a brief intervention involving goal setting was delivered by a dietitian, we would not have considered this to require the specialist knowledge of a dietitian but could have been feasibly delivered by other staff in primary care or community settings.

**Table 1:** Review eligibility criteria using PICOS framework.

	Inclusion criteria	Exclusion criteria
Population (P)	Women from pregnancy and up to 5-years after birth of the baby Women with or without health conditions Women who are or are not breast or chest feeding	Children from birth to age 5 (unless they are co-recipients of interventions targeting women in this time)
Intervention (I)	Intervention providers: non-specialist primary or community healthcare workforce including: Professionals working in contracted primary care providers (e.g., GPs, pharmacists, opticians, practice nurses, dentists) Allied health professionals (AHPs)^*^ Other ‘non-registered’ workforce, e.g., social prescribers/link workers, health care assistants, health trainers, practice managers and receptionists Health visitors Midwives Trialists/researchers without nutrition/weight management qualifications AND Non-specialist intervention type: Lower-intensity interventions, i.e., ≤1 session/month (11) e.g., brief advice, weight management counselling, referral or signposting to weight management support, measuring postnatal weight, motivational interviewing. Interventions not required to be delivered by staff with specialist nutrition/dietetic or weight management qualifications.	Interventions delivered during pregnancy only. Purely digital interventions. Dedicated weight management programmes, e.g., weight management programmes delivered by commercial organisations. Interventions required to be delivered by staff with formal specialist nutrition/dietetics training (e.g., dietitian).
Comparison (C)	Standard care. No intervention. Alternative intervention. No comparison (i.e., single-arm studies).	NA
Outcomes (O)	Intervention recipients: Maternal weight-related outcomes and/or behavioural outcomes (diet, physical activity) up to 5-year postnatal. Process outcomes, e.g., intervention participation, intervention adherence. Experiences/acceptability of receiving the intervention. AND/OR Workforce: Intervention fidelity and adherence. Experiences/acceptability of delivering the intervention.	Outcomes during pregnancy only (e.g., gestational weight gain).
Study design (S)	Any study design including randomised- or quasi-randomised trials, natural experiments, service evaluations, cohort studies. Studies written in English language. Studies conducted in high-income countries (12). Qualitative or mixed methods studies that are directly linked to eligible interventions, including process evaluations.	Studies set in low- or middle-income countries. Qualitative research on the general topic of postnatal weight management which is not linked to any eligible intervention.

^*^Art therapists, music therapists, drama therapists, dietitians, occupational therapists, orthoptists, orthotists, paramedics, physiotherapists, podiatrists, practitioner psychologists, prosthetists, speech and language therapists. NA, not applicable.

### 2.3 Screening, data extraction and quality appraisal

The results from the database searches were imported into Endnote (v.20) (13) and de-duplicated. These records were exported to Rayyan, an online screening tool (14), for title and abstract screening, and subsequent full text screening. Articles were screened at title and abstract and full text screening stages independently and in duplicate by NH, MF, RK, KT, MP, LSR, EC, RA, GN, and MM who were blind to each other’s decisions. Any discrepancies in screening decisions were resolved through discussion between the reviewers, and if necessary arbitrated by a third reviewer.

A pre-developed data extraction form based on the template for intervention description and replication (TiDIER) (15) framework for quantitative studies and the COnsolidated criteria for REporting Qualitative research (COREQ) (16) framework for qualitative studies was created and piloted with the review team. Data were extracted independently by two reviewers (MF and RK) in duplicate and discrepancies were resolved through discussion. Extracted data included relevant study information (study design, research aims, population and country), methods (eligibility criteria, recruitment method, randomisation procedure, intervention description, who delivered the intervention, setting, study dates and sample size estimation), analytical methods (outcomes, time points outcomes were assessed, analysis methods, statistical test, confounding factors, handling of missing data).Participant information (ethnic groups, age, number of pregnancies, parity, weight, BMI, breastfeeding, other socio-economic information), and results pertaining to each eligible outcome were also extracted.

The quality of the individual studies was assessed using the Joanna Briggs Institute (JBI) appraisal tools for quantitative (RCTs and quasi-experimental appraisal tool) and qualitative studies (17). Discrepancies were resolved through discussions among reviewers. The RCT quality appraisal tool provides questions that are assessed at a study level (i.e., overall) and outcome level (i.e., individual outcomes). When assessing RCTs we did not consider two of the signalling questions: *“were participants blind to treatment assignment?”* and *“were those delivering the treatment blind to treatment assignment?”* This decision was made as it is unlikely such an intervention could be blinded from the participant or the interventionist. All other signalling questions were considered. Additionally, the RCT quality appraisal tool has reporting bias related to each outcome individually. Since all outcomes were rated the same, we only provide a singular overall statement per signalling question.

### 2.4 Evidence synthesis

A descriptive overview of the characteristics of the included studies and participants is presented using narrative summaries and tables with key information. Quantitative outcome data were deemed too heterogeneous to pool in a meta-analysis due to the variation in reporting, assessment timing and intervention implementation periods. Results were, therefore, synthesised narratively. Tables were thematically produced according to the type of outcome being reported (e.g., effectiveness of the interventions on weight-related outcomes) with specific outcomes clustered within the tables (e.g., postnatal weight retention, weight loss, BMI change) to facilitate the synthesis of patterns in the data. Results are reported under the categories of intervention effectiveness, intervention implementation, and intervention experiences.

## 3 Results

### 3.1 Search results

**Figure 1:**
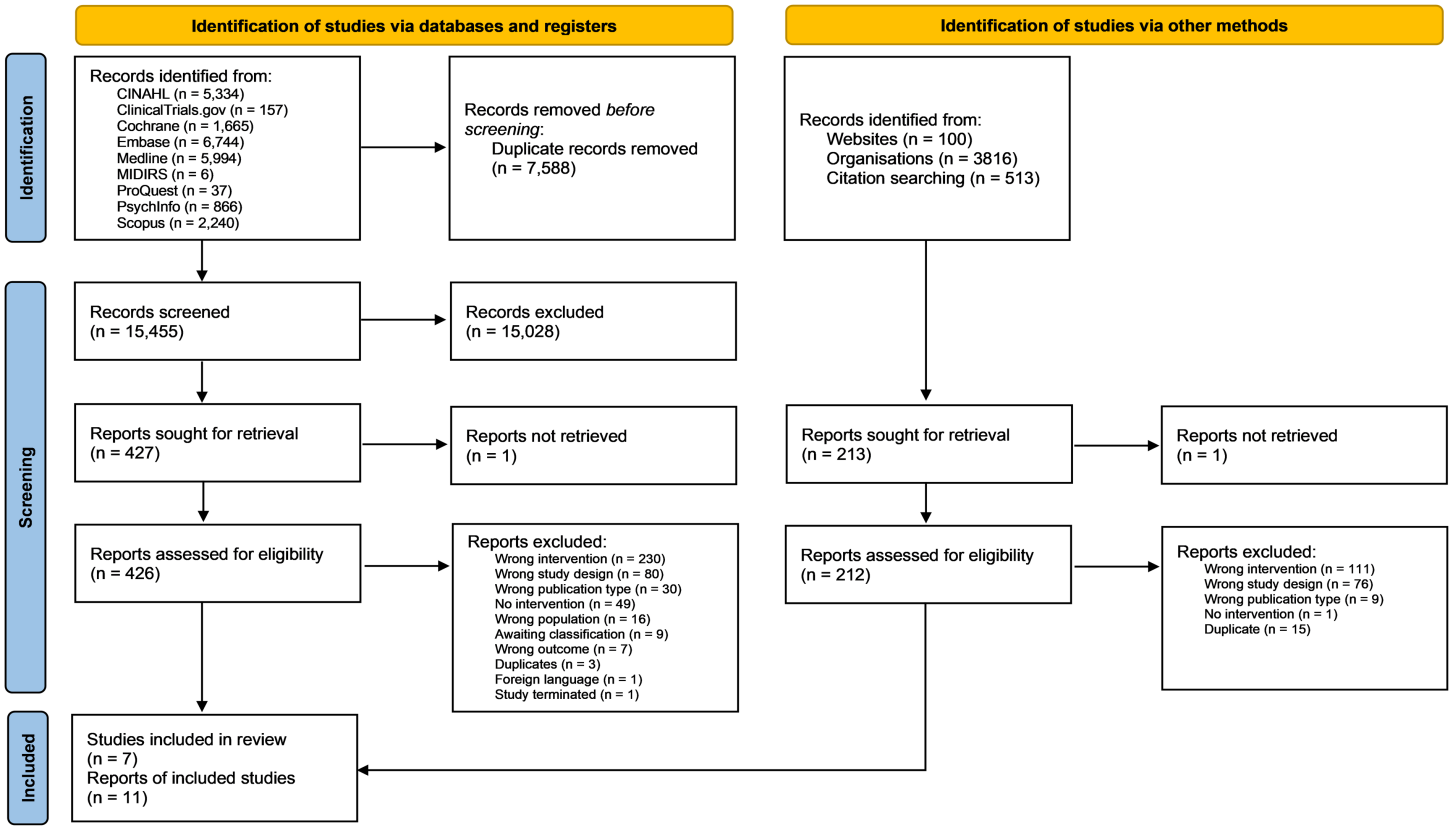
PRISMA flow diagram showing the flow of studies throughout the selection process.

The database searches identified 15,455 records for title and abstract screening after duplicates had been removed. A further 513 references and citations were screened from citation chaining and 3,916 from grey literature sources (see Figure 1). A total of seven studies described in 11 reports met the eligibility criteria for inclusion in the review.

### 3.2 Study characteristics

Study characteristics are shown in Table 2. Two studies were conducted in the Netherlands (18, 19), and one each in the United Kingdom (20), Germany (22), Taiwan (26), Finland (27), and the United States (28). Two studies were RCTs (18, 26), two were cluster RCTs (20, 22), one used a non-randomised, controlled cluster trial design (27), one a pre/post controlled study design (19), and one was a secondary analysis of a non-RCT (28). The median sample size was 240 participants and ranged from 28 (20) to 2,261 (22) participants. The mean age of participants was similar across all studies, ranging from 29 to 32 years old. Of the four studies that reported ethnicity or nationality (19, 20, 22, 28), participants were predominately white with inconsistent reporting of other ethnic groups. Two studies explicitly included primiparous women only (18, 27). Four studies reported pre-pregnancy or first trimester BMIs (18, 22, 26, 28). The remaining three studies reported baseline BMI at varying postnatal time points (1.5–6 months) (19, 20, 27). Two studies included women who were, on average, classified as having obesity (i.e., BMI ≥ 30 kg/m^2^) (20, 28).

**Table 2:** Characteristics of included studies.

Author (year) country	Research aim	Study design, sample size	Participant timepoint of study inclusion	Participant age [years mean (SD)]	Participant BMI [kg/m^2^ mean (SD)] at study inclusion	Inclusion criteria	Exclusion criteria
Althuizen et al. (2013) (18) Netherlands	To evaluate the effects of a counselling intervention on excessive weight gain during pregnancy and postnatal weight retention.	RCT *N* = 246	Pregnant women within 14 weeks gestation	Intervention: 29.2 (3.8) Control: 30.4 (4)	Baseline BMI measured early pregnancy: Intervention = 24 (4.2) Control = 23.5 (3.8)	Expecting first child; able to read, write and speak Dutch; in the first 14 weeks of gestation.	None reported.
Berks et al. (2019) (19) Netherlands	To evaluate the feasibility of a lifestyle intervention program after complicated pregnancies by comparing the proportion of eligible women who completed the intervention to other lifestyle intervention programs.	Pre/post controlled study *N* = 206	Women 6-months postnatal	Intervention = 31.6 (4.2) Control = 30.7 (8.8)	Baseline BMI measured postnatal: Intervention = 28.3 (6.6) Control = 26.4 (5.4)	Women with pregnancies complicated by preeclampsia, fetal growth restriction and/or gestational diabetes mellitus; age 18 years or above; Dutch, Turkish or Moroccan ethnicity; mastery of Dutch language.	Women with pre-pregnancy cardiovascular and/or metabolic conditions, pre-existent hypertension, or diabetes mellitus.
Daley et al. (2021) (20) and Daley et al. (2020) (21) United Kingdom	To produce evidence of whether a Phase III trial of a brief weight management intervention, in which postnatal women are encouraged by practice nurses as part of the national child immunisation programme to self-monitor their weight and use an online weight management programme, is feasible and acceptable.	Cluster RCT *N* = 28	Women 2-months postnatal	Overall = 32.1 (5.7) Intervention = 32.9 (6.1) Control = 31 (5.3)	Baseline BMI measured postnatal: Overall = 31.8 (6.9) Intervention = 31.6 (6.1) Control = 32.1 (8)	18+ years, given birth at least 4 weeks previously, registered as a patient at one of the participating practices, planning to have their child immunised and not yet attended the first appointment, BMI of 25+ at baseline home visit, able and willing to provide informed consent.	Baby had died or been removed from their care at birth, actively involved in a weight loss programme or trial, unwilling for researchers to notify GP about participation in trial, diagnosed with a serious mental health difficulty requiring hospitalisation in the past 2 years or anorexia/bulimia in the past 2 years
Hoffman et al. (2019) (22), Hoffman et al. (2020) (23), Kunath et al. (2019) (24), and Rauh et al. (2014) (25) Germany	To investigate the effect of the GeliS (“healthy living in pregnancy”) intervention on short- and long-term maternal PPWR and on the women’s breastfeeding behaviour assessed in a 12-month follow-up.	Cluster RCT *N* = 973	Pregnant women before the end of the 12th week of gestation.	Overall = 30.3 (4.4) Intervention = 30.2 (4.3) Control = 30.5 (4.6)	Baseline BMI measured early pregnancy: Overall = 24.4 (4.5) Intervention = 24.4 (4.3) Control = 24.3 (4.6)	Pre-pregnancy BMI between 18.5 and 40, singleton pregnancy, aged 18 to 43 years, sufficient German language skills, pregnant and before the end of the 12th week of gestation.	Multiple pregnancy, high risk pregnancy prohibiting study participation, pre-pregnancy early gestational diabetes mellitus uncontrolled chronic diseases, psychiatric or psychosomatic diseases, any other diseases which could prohibit study compliance.
Huang et al. (2011) (26) Taiwan	To examine and compare the effect of individual counselling about diet and physical activity on childbearing women during two periods: from pregnancy through to 6 months postnatal (intervention 1), and from birth through to 6 months postnatal (intervention 2).	RCT *N* = 240	Intervention 1: Pregnant women at 16 weeks gestation. Intervention 2: postnatal women 24–48 h after delivery.	Intervention 1 = 32.13 (4.5) Intervention 2 = 30.67 (3.7) Control = 31.91 (4.85)	Baseline BMI measured early pregnancy: Intervention 1 = 20.98 (2.34) Intervention 2 = 20.96 (3.22) Control = 21.12 (3.13)	18+ years, no cognitive impairment or psychiatric illness, ability to speak and read Chinese, not participating in another study, and intention to give birth at the study site.	None reported.
Kinnunen et al. (2007) (27) Finland	To investigate whether individual diet and physical activity counselling after pregnancy has positive effects on diet and leisure time physical activity (LTPA) and increases the proportion of primiparas who return to their pre-pregnancy weight by 10 months postnatal.	Cluster controlled trial (not randomised) *N* = 92	Women 2-months postnatal.	Intervention: 29.5 (3.9) Control: 28.3 (4.4)	Baseline BMI measured postnatal: Intervention = 24.3 (3.8) Control = 23.6 (2.5)	Primiparas; 18+ years.	<18 years, type 1 or 2 diabetes, twin pregnancy, physical disability preventing exercise, otherwise problematic pregnancy, substance abuse, treatment or clinical history of psychiatric illness, inadequate Finnish language, intention to change residence within 3-months.
Lohr et al. (2021) (28) United States	To evaluate the impact of daily self-weighing alone (without daily health advice on nutrition or weight loss) on postnatal weight loss.	Non-randomised controlled trial (secondary analysis) *N* = 428	At delivery	Intervention = 31.2 (4.92) Control = 31 (5.45)	Pre-pregnancy BMI Intervention = 31.8 (10.2) Control = 30.6 (9.63) Delivery BMI Intervention = 37 (8.78) Control = 35.7 (7.62)	Women admitted to the labour and delivery unit of a Midwestern academic hospital, 18+ years, with one of the following hypertension-related diagnoses during pregnancy: chronic, gestational, pre-eclampsia, or eclampsia, or a new hypertension diagnosis postnatal (29).	Readmitted to hospital after their primary hospital admission for delivery of their neonate.

BMI, body mass index; GDM, gestational diabetes mellitus; RCT, randomised controlled trial; SD, standard deviation.

### 3.3 Intervention characteristics

Intervention characteristics of included studies are presented in Supplementary Table S1.

#### 3.3.1 Intervention timing

In four studies, the intervention was delivered in the postnatal period exclusively – herein referred to as ‘Postnatal Only’ interventions (19, 20, 27, 28). These were initiated immediately after delivery (28), 2-months postnatal (20, 27), and 7-months postnatal (19). In two studies, intervention delivery started during pregnancy and involved one session at 6–8 weeks in the postnatal period – herein referred to as ‘Pregnancy + Postnatal’ interventions (18, 22). One study (26) had two intervention arms whereby one received a longer intervention during pregnancy through to 3-months postnatal (Pregnancy + Postnatal), and the other received a shorter intervention starting after delivery through to 3-months postnatal (Postnatal Only). The number of intervention sessions delivered in the postnatal period ranged from one (18, 22) to five (27); the timeframe of intervention completion ranged from 6-weeks postnatal (18) to 10-months postnatal (19, 27).

#### 3.3.2 Intervention delivery setting

Four interventions were delivered during routine healthcare contacts (20, 22, 26, 27) including child immunisation visits (20, 27) and antenatal/postnatal clinic visits (22, 26) and delivered by nurses, midwives and other members of the medical team such as gynaecologists. Most interventions were predominately delivered face-to-face, although in the study by Althuizen et al. (18) the postnatal intervention was delivered by telephone, and in the study by Berks et al. (19) telephone counselling was available to women who preferred it.

#### 3.3.3 Intervention content

Six interventions involved counselling on diet and physical activity in sessions lasting 15–45 min (18, 19, 22, 26, 27). The study by Berks et al. (19) was also supplemented by use of computer-based health education programme. In the study by Daley et al. (20) women in the intervention group were provided with brief advice about weight management, signposted to a more intensive online weight management programme, and encouraged to self-monitor their weight through weekly self-weighing. The study by Lohr et al. (28) involved daily self-weighing without provision of counselling. In all studies, the control group received usual care.

### 3.4 Outcomes

All included studies reported weight-related outcomes, and fewer reported additional outcomes (Supplementary Table S2). Three reported women’s diet and/or physical activity behaviours (20, 26, 27) and data related to implementation (19, 20, 26), two studies reported participant experiences of the intervention (quantitative survey (19) and qualitative interviews (20)), and only one study reported provider experiences (qualitative interviews) (20).

### 3.5 Risk of bias

Risk of bias assessment for RCTs (*n* = 4; Supplementary Figure S1) showed that two studies had low risk of bias due to randomisation processes (18, 20), while two were unclear (22, 26). Concealment of allocation was only explicitly reported in one study (20). One study was unclear with regards to the control arm content (18). Two studies reported blinding assessors to treatment group (18, 26) and one explicitly stated they did not (20). Three studies measured outcomes in a reliable manner (18, 20, 22), one was unclear (26). One study conducted an intention-to-treat (ITT) analysis (18). One study reported a power calculation for a MANOVA which is not reported in the results, and did not consider confounding factors in the analysis (26).

Studies that utilised a quasi-experimental design (*n* = 3) were all considered low risk of bias for identifying the cause and effect (Supplementary Figure S2). All studies were deemed low risk of bias for measurements being measured in the same way and reliably. Two had no concerns regarding the groups being dissimilar at baseline (27, 28). All were at high risk of bias for the lack of multiple measurements of the outcome (19, 27, 28). However, one did collect two post measures but not all analyses included the first follow up time point (5-months postnatal) and focused on the final follow up time point (10 months postnatal) (27). One study did not adequately describe their follow up, specifically, lacking detail for dropouts (28). One study had inadequate statistical analysis as they did not control for confounding factors (28).

The one qualitative study had some concerns regarding the congruity between the philosophical perspective and the research methodology (20). However, there was congruity between the research objectives, methods, analysis, and interpretation of the results. Whilst there was a statement included about the researchers’ experience, the reflexivity account was limited.

### 3.6 Intervention effectiveness

#### 3.6.1 Weight-related outcomes

Outcomes relating to weight were reported in all the included studies (*n* = 7) but varied in terms of the outcomes assessed. These included weight change, PPWR and various measures of anthropometry.

##### 3.6.1.1 Weight change

Weight change was reported by six studies (18–20, 22, 26, 28) with follow-up ranging from 1.5 months to 13-months postnatal and results were mixed. Three studies showed a reduction in weight among intervention groups (19, 20, 26) while three showed no difference (18, 22, 28) (Table 3). Neither Althuizen et al. (18) nor Hoffman et al. (22) (both Pregnancy + Postnatal) found a difference between the intervention and control group in weight change from early pregnancy to 12-months (mean difference (MD) 0.94 kg (95% CI 2.41–0.53) for Althuzien et al.; MD −0.69 kg (95%CI −1.57–0.19) for Hoffman *et al*). Although, in the study by Hoffman et al. (22) weight loss since delivery was significantly different between the intervention and control group at 12-months postnatal (adjusted MD 0.85 kg, 95%CI 0.22–1.49; *p* = 0.008).

**Table 3:** Effects of the interventions on weight change (kg).

Author (year) country	Intervention	Time points	Intervention kg mean (SD); sample size	Control kg mean (SD); sample size	Result statistics	Evidence summary
Althuizen et al. (2013) (18) Netherlands	4× face-to-face antenatal counselling sessions. 1× PN telephone call.	Pre-pregnancy^*^ (baseline)	67.4 (11.6); *n* = 106	68.6 (71.4); *n* = 113	NR	↔
		6.5 Months PN^$^	72.9 (14.9); NR	71.1 (13.3); NR	NR	
		12 Months PN^$^	71.1 (15); *n* = 96	69.7 (12.9); *n* = 92	Mean change interventio*n* = −1.75 kg (5.1) Mean change control = −0.53 kg (5.5) Between groups: Beta = 0.94 kg (95% CI: −2.41 to 0.53)	
Berks et al. (2019) (19) Netherlands	3× PN counselling sessions (preferably face-to-face), supported by computer-tailored health programme and questionnaires.	6 Months PN (baseline)	81 (20); *n* = 144	75 (16); *n* = 62	*p* = 0.02	↓
		13 Months PN	77 (19)	NR	Within group mean change: Model 1^a^ = −1.8 kg (95% CI: −3.2 to −0.3) Model 2^b^ = −1.9 kg (95% CI: −3.4 to −0.3)	
Daley et al. (2021) (20) and Daley et al. (2020) (21) United Kingdom	3× face-to-face PN brief counselling at child immunisation appointment with signposting to POWeR online tool and self-weigh weekly.	1.5 Months PN^$^ (baseline)	81.6 (13.7); *n* = 16	86.2 (21.2); *n* = 12	NR	↓
		4.5 Months PN^$^	78.3 (13.5); *n* = 15	88.1 (23.9); *n* = 12	Adjusted mean difference between groups^c^ = −7.5 kg (95% CI: −13.8 to −1.3)	
Hoffman et al. (2019) (22), Hoffman et al. (2020) (23), Kunath et al. (2019) (24), and Rauh et al. (2014) (25) Germany	3× face-to-face antenatal counselling sessions. 1× face-to-face PN counselling session.	<12 Weeks gestation (baseline)	69.8 (13.1); *n* = 973	68.9 (13.9); *n* = 934	NR	↔
		12 Months PN	69.7 (13.7); *n* = 841	69.8 (14.4); *n* = 828	Between groups: Unadjusted = −0.26 kg (95% CI: −2.1–1.58) Adjusted^d^ = −0.04 kg (95% CI: −0.97–0.89)	
Huang et al. (2011) (26) Taiwan	Intervention 1: 3× face-to-face antenatal counselling sessions. 3x face-to-face PN counselling sessions. Intervention 2: 3× face-to-face counselling sessions, plus a brochure.	Pre-pregnancy	Intervention 1 = 52.8 (8.4); *n* = 61 Intervention 2 = 52.6 (6.6); *n* = 64	53.5 (9.4); *n* = 64	ANOVA, *p* = 0.8	↓
		6 Months PN	Intervention 1 = 55.2 (8.6); *n* = 61 Intervention 2 = 56.7 (6.1); *n* = 64	58.6 (10.2); *n* = 64	Group by time interaction: *F* = 10.82, *p* < 0.001 Group main effect: *F* = 1.61, *p* = NR Time main effect: *F* = 288.01, *p* < 0.001	
Lohr et al. (2021) (28) United States^	Instructed to take daily blood pressure and weight measurements.	Delivery	NR; *n* = 214	NR; *n* = 214	NR	↔
		1.5 Months PN	Median weight loss (IQR); *n* = 203 Overall = 10.4 (7.7–13.6) Obese = 9.1 (7.7–13.6) Breastfeeding = 10 (8.2–12.7) Weighed ≥ half of days = 10.9 (7.7–13.6)	Median weight loss (IQR); *n* = 173 Overall = 10.4 (7.7–13.6) Non-obese = 10.4 (7.7–12.7) Not breastfeeding = 10.4 (7.7–13.6) Weighed <half of days = 9.3 (6.3–13.2)	Overall: *p* = 0.8 Obese versus non-obese: *p* = 0.16 Breastfeeding vs. not breastfeeding: *p* = 0.37 Weighed ≥ half of days vs. weighed < half of days: *p* = 0.06.	

^*^Self-reported; ^$^Calculated from reported weeks; ^Study presented results in pounds and has been converted to kg; ^a^Adjusted for change in control group; ^b^Adjusted for change in controls, measure at intake, duration of breastfeeding, pre-eclampsia and severity, and gestational age at delivery; ^c^Adjusted for practice (random effect), two minimisation variables (GP size list and index of multiple deprivation), and baseline value; ^d^Adjusted for pre-pregnancy BMI and age, parity, gestational age at inclusion, time of the PN weight assessment. Direction of effect: ↔ no effect; ↓ decreased; ↑ increased; ↑↓ mixed results. Colour coding: amber does not favour either group; green favours intervention group; red favours control group. PN, postnatal; NR, not reported; CI, confidence interval; SD, standard deviation.

Berks et al. (19) and Daley et al. (both Postnatal Only) found a significant difference between intervention and control groups. The adjusted weight change attributed to the intervention was −1.9 kg (95%CI −4.3 to −0.3) at 13-months postnatal and −7.5 kg (95% CI: −13.8 to −1.3) at 4.5 months postnatal, respectively. Lohr et al. (28) (Postnatal Only) found no difference in weight change (*p* = 0.80) or percentage weight loss (*p* = 0.35) between the intervention and control group at 6-weeks postnatal (28).

In the study by Huang et al. (26), *post hoc* tests following ANOVA showed that the Pregnancy + Postnatal intervention had a significantly greater effect on weight change than both the Postnatal Only and control groups at 6-months postnatal.

##### 3.6.1.2 Postpartum weight retention

Four studies reported PPWR (18, 22, 26, 27). Three studies reported this as the proportion of women with PPWR ≥5 kg (18, 22, 26) (Table 4). Neither Althuizen et al. (18) [OR 1.20 (95% CI: 0.41–3.51)] nor Hoffman et al. (22) [adjusted OR 0.72 (95% CI: 0.47–1.11), *p* = 0.142] (both Pregnancy + Postnatal) found an intervention effect at 13- and 12-months postnatal, respectively. Huang et al. (26) observed significantly higher PPWR in the control group (51.6%) than both intervention groups (Pregnancy + Postnatal = 18%, Postnatal Only = 42.2%; *p* = 0.000). Similarly, in the study by Kinnunen et al. (27) (Postnatal Only) the intervention arm were significantly more likely to have PPWR ≤0 kg at 10-months postnatal compared to controls [OR 3.89 (95% CI: 1.16–13.04), *p* = 0.028] (27).

**Table 4:** Effects of the interventions on postnatal weight retention.

Author (year) country	Intervention	Outcome definition	Timepoints	Intervention; sample size	Control; sample size	Results	Evidence summary
Althuizen et al. (2013) (18) Netherlands	4x face-to-face antenatal counselling sessions. 1x PN telephone call.	Substantial weight retention >5 kg from 15 weeks of gestation to 13 months PN; percentage	13 Months PN^$^	19.4%; *n* = 19/96^*^	12.2%; *n* = 11/92^*^	Between groups, odds ratio^a^ = 1.20 (95% CI: 0.41–3.51)	↔
Hoffman et al. (2019) (22), Hoffman et al. (2020) (23), Kunath et al. (2019) (24), and Rauh et al. (2014) (25) Germany	3x face-to-face antenatal counselling sessions. 1x face-to-face PN counselling session.	Weight retention >5 kg from before 12 weeks of gestation to 12-months PN; percentage	12 Months PN	11.4%; *n* = 96/843	14.8%; *n* = 123/832	Between groups, odds ratio: Unadjusted = 0.81 (95% CI: 0.55–1.19), *p* = 0.277 Adjusted^b^ = 0.72 (95% CI: 0.47–1.11), *p* = 0.142	↔
		Weight retention KG; Mean (SD)		−0.2 (4.8); *n* = 860	0.6 (5.2); *n* = 848	Between groups, mean difference: Unadjusted = −0.63 kg (95% CI: −1.44–0.19), *p* = 0.132 Adjusted^b^ = −0.69 kg (95% CI: −1.57–0.19), *p* = 0.123 *Post hoc* adjustment for exclusive breastfeeding = −0.74 kg (95% CI: −1.55–0.07), *p* = 0.075.	
Huang et al. (2011) (26) Taiwan	Intervention 1: 3× face-to-face antenatal counselling sessions. 3× face-to-face PN counselling sessions. Intervention 2: 3× face-to-face counselling sessions, plus a brochure.	Weight retention >5 kg from 16 weeks gestation to 6 months PN; percentage	6 Months PN	Intervention 1: 18%; *n* = 11/61 Intervention 2: 42.2%; *n* = 27/64	51.6%; *n* = 33/64	Chi-square = 15.85, *p* = 0.000	↓
		Weight retention KG; mean (SD)		Intervention 1: 2.34 (2.66); *n* = 61 Intervention 2: 4.06 (3.6); *n* = 64	5.08 (3.32); *n* = 64	Between groups: *F* = 11.43, *p* < 0.001 Intervention 1 significantly less than intervention 2 and control.	
Kinnunen et al. (2007) (27) Finland	4× face-to-face PN counselling sessions at child clinical visits.	Weight retention of ≤0 kg from pre-pregnancy to 10 months PN; percentage	10 Months PN	50%; *n* = 23/46	30%; *n* = 11/37	Between groups: Chi-square, *p* = 0.06 Odds ratio^c^^ = 3.89 (95% CI: 1.16–13.04), *p* = 0.028	↑↓
		Weight retention KG; mean (SD)		1.8 (4.3); *n* = 46	1.0 (4.4); *n* = 37	Adjusted mean difference^d^ = 0.8 kg (95% CI: −1.1 to 2.7), *p* = 0.42.	

^$^Calculated from reported weeks; ^*^Calculated from available percentage; ^Number of participants is less in analysis, intervention = 43 and control = 35 percentage not reported; ^a^Adjusted for age, education and pre-pregnancy BMI; ^b^Adjusted for pre-pregnancy BMI and age, parity, gestational age at inclusion, time of the pp weight assessment; ^c^Adjusted for age, education, pre-pregnancy BMI, gestational weight gain, weight at 2 months postnatal (baseline), duration of exclusive breastfeeding and smoking status; ^d^Adjusted for pre-pregnancy weight. Direction of effect: ↔ no effect; ↓ decreased; ↑ increased; ↑↓ mixed results. Colour coding: amber does not favour either group; green favours intervention group; red favours control group. PN, postnatal; CI, confidence interval.

Three studies reported mean differences for PPWR in kilograms (22, 26, 27). Hoffman et al. (22) (Pregnancy + Postnatal) reported no significant differences between the intervention and control groups in adjusted and unadjusted analyses. Huang et al. (26) reported lower PPWR in the Pregnancy + Postnatal arm compared to the Postnatal Only and control groups (*p* < 0.001). Kinnunen et al.’s (27) (Postnatal Only) intervention also reported no significant difference [adjusted MD 0.8 kg (95% CI: −1.1 to 2.7), *p* = 0.42].

##### 3.6.1.3 Other weight-related changes

Among the studies reporting other weight-related outcomes (Table 5), Daley et al. (20) and Berks et al. (19) (both Postnatal Only) reported significant reductions in BMI in the intervention groups compared with control groups at 4.5 months [adjusted MD = −3.1 kg/m^2^ (95%CI −5.8 to −0.3 kg/m^2^)] and 13-months postnatal [adjusted MD = −0.9 kg/m^2^ (95% CI −1.4 to −0.3 kg/m^2^)], respectively. These studies also reported a significant reduction in body fat percentage (20) and waist-to-hip ratio (19) in the intervention groups. However, Kinnunen et al. (27) (Postnatal Only) reported no significant difference in waist circumference between groups at 10-months postnatal (*p* = 0.24).

**Table 5:** Effects of the interventions on body fat percentage, BMI, waist circumference and waist-to-hip ratio.

Author (year) country	Intervention	Outcome definition	Time points	Intervention; sample size	Control; sample size	Results	Evidence summary
Berks et al. (2019) (19) Netherlands	3× PN counselling sessions (preferably face-to-face), supported by a computer-tailored health programme and questionnaires.	Body mass index (BMI) kg/m^2^; mean (SD)	6 Months PN (baseline)	28.3 (6.6); *n* = 144	26.4 (5.4); *n* = 62	*p* = 0.05	↓
			13 Months PN	27 (6); *n* = 144	NR	Within group: Model 1^a^ = −0.8 kg/m^2^ (95% CI: −1.3 to −0.3 kg/m^2^) Model 2^b^ = −0.9 kg/m^2^ (95% CI: −1.4 to −0.3 kg/m^2^)	
		Waist-to-hip ratio cm/cm; mean (SD)	6 Months PN (baseline)	0.83 (0.06); *n* = 144	0.82 (0.06); *n* = 62	*p* = 0.14	
			13 Months PN	0.81 (0.07); *n* = 144	NR	Within group: Model 1^a^ = −0.04 cm/cm (95% CI: −0.06 to −0.03 cm/cm) Model 2^b^ = −0.04 cm/cm (95% CI: −0.06 to −0.03 cm/cm)	
Daley et al. (2021) (20) and Daley et al. (2020) (21) United Kingdom	3× face-to-face PN brief counselling at child immunisation appointment with signposting to POWeR online tool and self-weigh weekly.	Body Fat percentage (%); mean (SD)	1.5 Months PN^$^ (baseline)	40.9 (4); *n* = 16	41.6 (6); *n* = 12	NR	↓
			4.5 Months PN^$^	39.6 (4.7); *n* = 15	42.4 (7.1); *n* = 12	Between groups: Adjusted^c^ = −3.2% (95% CI: −6.3 to −0.1%)	
		Body Mass Index (BMI) kg/m^2^; mean (SD)	1.5 Months PN^$^ (baseline)	31.6 (6.1); *n* = 16	32.1 (8); *n* = 12	NR	
			4.5 Months PN^$^	30.2 (6); *n* = 15	32.8 (8.8); *n* = 12	Between groups: Adjusted = −3.1 kg/m^2^ (−5.8 to −0.3 kg/m^2^)	
Kinnunen et al. (2007) (27) Finland	4x face-to-face PN counselling sessions at child clinical visits.	Waist circumference (cm); mean (SD)	2 Months PN (baseline)	81.8 (9); *n* = 48	81.1 (6.7); *n* = 37	*p* = 0.66	↔
			10 Months PN	78.1 (10.2); *n* = 46	75.4 (6.2); *n* = 37	Between groups: Adjusted^d^ = 1.0 cm (95% CI: 0.7–2.7), *p* = 0.24	

^$^Calculated from reported weeks; ^a^Adjusted for change in control group; ^b^Adjusted for change in controls, measure at intake, duration of breastfeeding, pre-eclampsia and severity, and gestational age at delivery; ^c^Adjusted for practice (random effect), two minimisation variables (GP size list and index of multiple deprivation), and baseline value; ^d^Adjusted for pre-pregnancy weight. Direction of effect: ↔ no effect; ↓ decreased; ↑ increased; ↑↓ mixed results. Colour coding: amber does not favour either group; green favours intervention group; red favours control group. PN, postnatal; CI, confidence interval; kg/m^2^, kilogram per metre-squared; cm, centimetres; SD, standard deviation.

#### 3.6.2 Behaviour-related outcomes

Four studies reported changes related to physical activity (Table 6) and dietary outcomes (19, 20, 26, 27) (Table 7).

**Table 6:** Effects of the interventions on physical activity outcomes.

Author (year) country	Intervention	Outcome definition and assessment tool	Timepoints	Intervention; sample size	Control; sample size	Results	Evidence summary
Berks et al. (2019) (19) Netherlands	3x PN counselling sessions (preferably face-to-face), supported by a computer-tailored health programme and questionnaires.	MET (IPAQ); Mean (SD)	6 Months PN (baseline)	3,672 (6554); *n* = 144	NR; *n* = 62	NR	↔
			13 Months PN	3,830 (4240); *n* = 144	NR	Within group: Model 1^a^ = 2,251 (95% CI: 329–4,174) Model 2^b^ = 844 (95% CI: −945–2,634)	
		Steps per day (IPAQ); Mean (SD)	6 Months PN (baseline)	8,290 (2508); *n* = 144	NR; *n* = 62	NR	
			13 Months PN	8,658 (2099); *n* = 144	NR	Within group: Model 1^a^ = 52 (95% CI: −695–1,599) Model 2^b^ = 302 (95% CI: −1,373–770)	
Daley et al. (2021) (20) and Daley et al. (2020) (21) United Kingdom	3x face-to-face PN brief counselling at child immunisation appointment with signposting to POWeR online tool and self-weigh weekly.	Total activity, MET hours per week (PPAQ); Median (IQR)	1.5 Months PN^$^ (baseline)	289.7 (224.2–416.2); *n* = 13	345.6 (328.1–423.1); *n* = 11	NR	↔
			4.5 Months PN^$^	265 (224.8–434.6); *n* = 12	278.6 (212.8–409.7); *n* = 11	Between group: Unadjusted = −13.2 (95% CI: −209.1–182.7)	
Huang et al. (2011) (26) Taiwan	Intervention 1: 3x face-to-face antenatal counselling sessions. 3x face-to-face PN counselling sessions. Intervention 2: 3x face-to-face counselling sessions, plus a brochure.	Healthy promoting behaviour physical activity (Health Promoting Lifestyle Profile – Chinese version); Mean (SD)	Intervention 1 = 16 weeks gestation (baseline) Intervention 2 = 24–48 h post birth (baseline)	Intervention 1 = 8.52 (2.31); *n* = 61 Intervention 2 = 9.16 (2.22); *n* = 64	9.06 (2.17); *n* = 64	NR	↑
			6 Months PN	Intervention 1 = 10.97 (1.92); *n* = 61 Intervention 2 = 10.84 (2); *n* = 64	9.34 (2.51); *n* = 64	*F*-value: Interaction effect = 13.29, *p* < 0.001 Group main effect = 3.10, *p* < 0.05 Time main effect = 71.83, *p* < 0.001	
		Self-efficacy physical activity(Self-Rated Abilities for Health Practices Scale – Chinese version); Mean (SD)	Intervention 1 = 16 weeks gestation (baseline) Intervention 2 = 24–48 h post birth (baseline)	Intervention 1 = 15.05 (5.77); *n* = 61 Intervention 2 = 14.53 (5.58); *n* = 64	14.22 (5.96); *n* = 64	NR	
			6 Months PN	Intervention 1 = 22.08 (3.57); *n* = 61 Intervention 2 = 19.2 (3.89); *n* = 64	18.31 (2.91); *n* = 64	*F*-value: Interaction effect = 3.59, *p* < 0.05 Group main effect = 7.21, *p* < 0.001 Time main effect = 125.16, *p* < 0.001	
Kinnunen et al. (2007) (27) Finland	4x face-to-face PN counselling sessions at child clinical visits.	MET minutes per week (modified IPAQ); Mean (SD)	2 Months PN (baseline)	2,328 (1308); *n* = NR	2,601 (975); *n* = NR	NR	↔
			10 Months PN	1906 (970); *n* = NR	2051 (1249); *n* = NR	Adjusted^c^ = NS	

^$^Calculated from reported weeks; ^a^Adjusted for change in control group; ^b^Adjusted for change in controls, measure at intake, duration of breastfeeding, pre-eclampsia and severity, and gestational age at delivery; ^c^Adjusted for baseline weekly METmin, age, education, gestational weight gain and BMI at 2 months postnatal. Direction of effect: ↔ no effect; ↓ decreased; ↑ increased; ↑↓ mixed results. Colour coding: amber does not favour either group; green favours intervention group; red favors control group. IPAQ, International Physical Activity Questionnaire; MET, Metabolic Equivalent of Task; PPAQ, Pregnancy Physical Activity Questionnaire; NR, not reported; NS, not significant.

**Table 7:** Effects of the interventions on diet-related outcomes.

Author, country	Intervention	Outcome definition and assessment tool	Time points	Intervention; sample size	Control; sample size	Results	Evidence summary
Berks et al. (2019) (19) Netherlands	3× PN counselling sessions (preferably face-to-face), supported by a computer-tailored health programme and questionnaires.	Fat intake g/day (Maastricht Fatlist); Mean (SD)	6 months PN (baseline)	17 (5.1); *n* = 144	NR; *n* = 62	NR	↑↓
			13 months PN	15.1 (5.1); *n* = 144	NR	Within group: Model 1^a^ = −2.9 (95% CI: −4.7 to −1) Model 2^b^ = −2.9 (95% CI: −4.6 to −1.2)	
		Fat intake snacks g/day (Maastricht Fatlist) Mean (SD)	6 months PN (baseline)	6.2 (3); *n* = 144	NR; *n* = 62	NR	
			13 months PN	5.5 (3); *n* = 144	NR	Within group: Model 1^a^ = −0.8 (95% CI: −2 to −0.4) Model 2^b^ = −1 (95% CI: −2 to 0.04)	
Daley et al. (2021) (20) and Daley et al. (2020) (21) United Kingdom	3x face-to-face PN brief counselling at child immunization appointment with signposting to POWeR online tool and self-weigh weekly.	Eating behaviour (TFEQ); Mean (SD)	1.5 months PN^$^ (baseline)	Cognitive restraint = 38.7 (15) Uncontrolled eating = 47.9 (26.3) Emotional eating = 47.9 (26.5) *N* = 16	Cognitive restraint = 44.1 (28.1) Uncontrolled eating = 43 (23.7) Emotional eating = 48.5 (32.7) *N* = 11	NR	↑↓
			4.5 months PN^$^	Cognitive restraint = 47.6 (12.7) Uncontrolled eating = 50.3 (25.6) Emotional eating = 56.3 (34.4) *N* = 14	Cognitive restraint = 48.6 (23.3) Uncontrolled eating = 41 (27.9) Emotional eating = 43.5 (32.3) *N* = 12	Between groups, adjusted: Cognitive restraint = 5.4 (−8.9–19.6) Uncontrolled eating = −0.03 (−15.4–15.4) Emotional eating = 9.1 (−25.9–44)	
		Dietary choices score (WCSS); Mean (SD)	1.5 months PN^$^ (baseline)	NR	NR		
			4.5 months PN^$^	2.3 (0.7); *n* = 13	2.5 (0.8); *n* = 12	Between group: Adjusted = −0.2 (95% CI: −0.8–0.3)	
Huang et al. (2011) (26) Taiwan	Intervention 1: 3x face-to-face antenatal counselling sessions. 3x face-to-face PN counselling sessions. Intervention 2: 3x face-to-face counselling sessions, plus a brochure.	Healthy promoting behaviour nutrition (Health Promoting Lifestyle Profile – Chinese version); Mean (SD)	Intervention 1 = 16 weeks gestation (baseline) Intervention 2 = 24-48 h post birth (baseline)	Intervention 1 = 26.28 (4.3); *n* = 61 Intervention 2 = 26.25 (4.4); *n* = 64	25.89 (4.47); *n* = 64	NR	↑
			6 Months PN	Intervention 1 = 29.07 (3.94); *n* = 61 Intervention 2 = 28.77 (4.09); *n* = 64	24.98 (4.03); *n* = 64	*F*-value: Interaction effect = 9.64, *p* < 0.001 Group main effect = 9.13, *p* < 0.001 Time main effect = 14.57, *p* < 0.001	
		Self-efficacy nutrition (Self-Rated Abilities for Health Practices Scale – Chinese version); Mean (SD)	Intervention 1 = 16 weeks gestation (baseline) Intervention 2 = 24-48 h post birth (baseline)	Intervention 1 = 9.46 (2.34); *n* = 61 Intervention 2 = 9.42 (2.72); *n* = 64	9.5 (2.77); *n* = 64	NR	
			6 Months PN	Intervention 1 = 12.11 (2.18); *n* = 61 Intervention 2 = 10.33 (2.02); *n* = 64	10.66 (2.21); *n* = 64	*F*-value: Interaction effect = 7.06, *p* < 0.001 Group main effect = 3.78, *p* < 0.05 Time main effect = 59.46, *p* < 0.001	
Kinnunen et al. (2007) (27) Finland	4x face-to-face PN counselling sessions at child clinical visits.	Vegetables, fruit and berries portions per day (FFQ); Mean (SD)	2 Months PN (baseline)	2.4 (1.3); *n* = 44	2.7 (2); *n* = 37	NR	↑↓
			5 Months PN	2.6 (1.4); *n* = 44	2.6 (1.8); *n* = 37	Between groups, Adjusted^d^ = 0.4 (95% CI: −0.1–0.9), *p* = 0.13	
			10 Months PN	2.6 (1.4); *n* = 44	2.5 (2.1); *n* = 37	Between groups, Adjusted^d^ = 0.2 (95% CI: −0.3–0.8), *p* = 0.42	
		High-fibre bread percentage of total bread (FFQ); Mean (SD)	2 Months PN (baseline)	49 (29); *n* = 44	49 (30); *n* = 37	NR				
			5 Months PN	60 (29); *n* = 44	45 (33); *n* = 37	Between groups, Adjusted^d^ = 16 (95% CI: 4.2–27.7), *p* = 0.008		
			10 Months PN	65 (27); *n* = 44	52 (31); *n* = 37	Between groups, Adjusted^d^ = 16.1 (95% CI: 4.3–27.9), *p* = 0.008	
		High-sugar snacks portions per day (FFQ); Mean (SD)	2 Months PN (baseline)	1.9 (1.2); *n* = 44	2 (1.2); *n* = 37	NR	
			5 Months PN	2.2 (1.3); *n* = 44	1.5 (0.9); *n* = 37	Between groups, Adjusted^d^ = 0.6 (0.1–1.1), *p* = 0.028	
			10 Months PN	2.1 (1.2); *n* = 44	2.1 (1.4); *n* = 37	Between groups, Adjusted^d^ = 0 (−0.6–0.6), *p* = 0.93	

^$^Calculated from reported weeks; ^a^Adjusted for change in control group; ^b^Adjusted for change in controls, measure at intake, duration of breastfeeding, pre-eclampsia and severity, and gestational age at delivery; ^c^Adjusted for baseline weekly METmin, age, education, gestational weight gain and BMI at 2 months postnatal; ^d^Adjusted for baseline intake of the outcome variable, age, education, smoking status, gestational weight gain and BMI at 2 months postnatal. Direction of effect: ↔ no effect; ↓ decreased; ↑ increased; ↑↓ mixed results. Colour coding: amber does not favour either group; green favours intervention group; red favours control group. FFQ, food frequency questionnaire; g/day, grams per day; TFEQ, Three Factor Eating Questionnaire; WCSS, Weight Control Strategies Score; PN, postnatal.

##### 3.6.2.1 Physical activity outcomes

Two studies (19, 27) measured physical activity using the International Physical Activity Questionnaire (IPAQ) (30) and one (20) used the Pregnancy Physical Activity Questionnaire (PPAQ) (31). Huang et al. (26) reported healthy promoting behaviour and self-efficacy, both of which included physical activity subscales from the Health-Promoting Lifestyle Profile (Chinese version) (32, 33) and Self-Rated Abilities for Health Practices Scale (Chinese Version) (34, 35).

Berks et al. (19), Daley et al. (20), and Kinnunen et al. (27) (all Postnatal Only) all reported no statistically significant changes in metabolic equivalent of task (MET) measures (19, 20, 27). Berks et al. also reported no difference in step count (19). Huang et al. (26) found that physical activity health promoting behaviours and self-efficacy measures favoured the Pregnancy + Postnatal group compared to the Postnatal Only and control groups.

##### 3.6.2.2 Diet related outcomes

Diet related outcomes were assessed using a multitude of measures. One study (27) used a food frequency questionnaire (36) (FFQ); one (19) used the Maastricht Fatlist (37); one (20) used the revised Three Factor Eating Questionnaire (TFEQ) (38); one (20) used a Weight Control Strategies Score (WCSS); and one (26) used the Healthy promoting behaviour (diet subscale) from the Health-Promoting Lifestyle Profile (32, 33) (Chinese version), and perceived self-efficacy for weight management (diet subscale) from the Self-Rated Abilities for Health Practices Scale (34, 35) (Chinese Version).

Huang et al. (26) found that nutrition health promoting behaviours and self-efficacy measures favoured the Pregnancy + Postnatal group compared to the Postnatal Only and control groups. Berks et al. (19), Daley et al. (20), and Kinnunen et al. (27) (all Postnatal Only) also reported diet-related outcomes. There were no differences between intervention and control groups reported for consumption of vegetables, fruits, and berries (27), TFEQ domains (cognitive restraint, uncontrolled eating, and emotional eating), or dietary choices (20). Kinnunen et al. (27), reported a significant increase in the proportion of high-fibre bread intake of total bread intake (27) at 10-months postnatal [adjusted MD = 16.1 (95% CI: 4.3–27.9), *p* = 0.008] and Berks et al. (19) reported reductions in total daily fat intake [adjusted MD = −2.9 g/day (95% CI −4.6 to −1.2)]. A significant reduction in snacks high in fat was also observed in Berks et al. (19), although this did not remain significant in adjusted analysis. Kinnunen et al. (27) also reported a significant increase in snacks high in sugar at 5 months postnatal in the intervention group compared with control [adjusted MD = 0.6 portions (95%CI 0.1–1.1), *p* = 0.028]; however, intake was similar at 10-months postnatal between the two groups [MD 0 portions (95%CI −0.6 to 0.6), *p* = 0.93].

### 3.7 Implementation outcomes

#### 3.7.1 Intervention and study drop-out

Drop-out rates at end of intervention could be extracted from five studies (19, 20, 22, 26, 27) and drop-out rates at last follow-up could be extracted from six (18–20, 22, 26, 27) (Supplementary Table S3). Drop-out rates at end of intervention ranged from 6.3% (20) to 28.0% (19). Drop-out rates at last-follow up ranged from 3.6% (20) to 38.8% (19) and tended to be similar between intervention and control groups within studies. One exception was the study by Berks et al. (19) in which 20 more participants in the control group withdrew. However, this was primarily due to more women in this group having a new pregnancy during the follow-up period [*n* = 18 out of 62 control participants (29.0%)] compared to the intervention group [*n* = 3 out of 144 intervention participants (2.1%)].

#### 3.7.2 Study and intervention process outcomes

Four studies reported intervention implementation and process outcomes (18–20, 28). Statistical tests were not conducted for any of these outcomes (Supplementary Table S4). Two studies reported similar results in relation to metrics of study participation, although these were operationalised differently. Daley et al. (20) reported recruitment rate as the proportion of women recruited (*n* = 28) out of the recruitment target (*n* = 80) and this was 35% (95% CI = 25–45%). Berks et al. (19) reported participation rate as the proportion of women who agreed to participate in the study (*n* = 144) out of total women who were eligible for participation (*n* = 407), and this was also 35%.

Intervention adherence was reported by all four studies and included both adherence to the intervention by participants (18–20, 28) and adherence to intervention delivery by the workforce (20). In the study by Althuizen et al. (18) most women in the intervention group (67%; *n* = 83) attended all five sessions including the one session in the postnatal period. In the study by Daley et al. (20) data from the bodytrace weighing scales showed that 63% (95% CI = 39–86%) (*n* = 10) adhered to weekly self-weighing, while 56% (95% CI = 32–81%) (*n* = 9) registered with the online POWeR programme. In the study by Lohr et al. (28), 77% (*n* = 165) of women were adherent to daily self-weighing (self-weighed ≥4 times/week) and the mean frequency of self-weighing was 4.8 times per week. Berks et al. (19) reported study adherence as the proportion of women who attended the 13-month visit out of women who agreed to participate, and this was 65% (*n* = 94) and 52% (*n* = 32) for the intervention and control groups, respectively. Intervention completion was reported as the proportion of participants who completed the intervention (*n* = 94) out of women who were eligible to take part in the intervention (*n* = 407) and this was 23%. In relation to workforce adherence to intervention delivery, Daley et al. (20) assessed this through consultation recordings (*n* = 17 recordings). Nurses adhered to weighing women and recording their weight (≥60% of the time) in 69% of recorded consultations, while checking that participants were self-weighing, signposting participants to POWeR and asking participants if they had accessed POWeR were each observed in 88.2% of recorded consultations.

### 3.8 Intervention experiences

Intervention experiences were reported by two studies for intervention participant perspectives (19, 20) and for one study for intervention delivery staff perspectives (20).

#### 3.8.1 Participant perspectives

Intervention participants in the study by Berks et al. (19) reported perceived barriers and motivators to engaging with the intervention and their satisfaction with the programme in bespoke questionnaires that were informed by previous qualitative work. Questionnaires were completed by 99% of the women who completed the intervention, but none of the women who dropped out. Authors reported that 86% of respondents were satisfied with the behavioural intervention and 89% were satisfied with counselling sessions. Satisfaction with the computer-tailored health education program was lower (61%). Perceived barriers to intervention participation were travel distance (33%) and travel time (35%) to the hospital, although 76% considered the hospital to be a good setting for the counselling sessions. A total of 65% agreed that counselling sessions conducted by telephone were a good alternative to face-to-face counselling. While authors reported that a qualitative semi-structured interview was conducted among women in the intervention group, findings were not reported.

Daley et al. (20) explored women’s experience of the intervention through semi-structured interviews (*n* = 9) after intervention completion. Analysis yielded three themes: barriers and facilitators to weight loss; evaluation of the trial; and feelings around weighing and weight loss. All interviewees found the immunisation clinic an appropriate setting for a weight loss intervention, and for nurses to refer them to a website rather than providing information themselves at the appointment. While women were generally unfazed by being weighed at the immunisation clinic, some reported fear of being weighed if they anticipated weight gain. Generally, women found nurses to be non-judgmental and encouraging. Women were receptive to self-weighing and recording of weight, although issues with the remote tracking digital scales made women question their reliability. Self-weighing elicited mixed emotions depending on anticipated weight change and helped some women to gain a sense of control over their weight. Women perceived the POWeR website to be a motivating source of trusted information (although some information had been seen before) and only a few women experienced difficulties in accessing it. Some women suggested the programme could be improved if it contained more information specific to the postnatal experience, and if it was more accessible by mobile phone.

#### 3.8.2 Staff perspectives

Daley et al. (20) conducted semi-structured interviews with practice nurses (*n* = 6) and a GP (*n* = 1) who delivered the intervention. Staff perceived that the intervention was generally a good idea; however, there were concerns that women did not need additional pressure to lose weight during an already challenging time with a new baby, and that raising the topic of weight may deter women from attending immunisation appointments. They also expressed concern about the time available during the visit to raise the topic of weight and provide adequate support, and the potential to damage the relationship with mother. While staff felt comfortable and confident weighing trial participants, they anticipated they would feel less confident weighing mothers as part of standard practice outside of a trial setting. They reported that most women were comfortable being weighed, although some were uncomfortable or embarrassed, and a few participants declined to be weighed, while some needed encouragement.

Notwithstanding initial nervousness, most staff felt prepared to deliver the intervention and that delivering the intervention was not onerous. While staff perceived that women were receptive to referral to the POWeR website, some were concerned about the digital literacy of participants and suggested that physical information should also be available, e.g., leaflets or person–person advice. Staff made several suggestions to improve the format and mode of intervention training, and some also suggested that the intervention was delivered to women either earlier (e.g., antenatally) or later (e.g., 12-weeks after delivery). Potential logistical challenges to wider intervention implementation were also raised, e.g., limited time available during visits to weigh women and record weight.

## 4 Discussion

### 4.1 Key findings

This systematic review aimed to determine the effectiveness and implementation of lower-intensity weight management support delivered by the non-specialist workforce to women up to 5 years postnatal. We identified seven unique studies reported in 11 publications that met our eligibility criteria. While some interventions showed an effect on postnatal weight, it is challenging to draw confident conclusions on the effectiveness and usefulness of these interventions due to limited evidence and heterogeneity in study design and reporting. The longest follow-up data were collected 13-months postnatal, far short of our time period of interest. Only two studies explored intervention acceptability and patient experience. To make any firm conclusions in relation to our research aim, further low-intensity interventions embedded in primary and community care settings are needed, with consistent outcome and process evaluation data collection to facilitate comparisons of studies across different populations and contexts.

### 4.2 Comparison with wider literature

Previous reviews that included higher-intensity interventions report 1.5–3.0 kg more weight loss in the intervention groups than controls (7–9, 39). In our review, the absolute weight change in both intervention and control groups could not be determined in several studies, making comparisons with previous literature challenging. Additionally, there was heterogeneity in the timing of the last follow-up assessment relative to the end of the intervention. For example, Althuizen et al. (18) conducted the last intervention session at 8-weeks postnatal and the final follow-up assessment was conducted at 13-months postnatal. Whereas in Daley et al. (20), the last intervention session and final assessment was conducted at 4.5-months postnatal. The studies where the final assessments were conducted in tandem with the last intervention session tended to appear more effective. This is expected given that participants in these studies were still actively engaged with the intervention and had recent contact with intervention provider at the time of their final assessment. Our findings vary somewhat from those of a 2018 systematic review and meta-analysis which found similar weight loss regardless of whether weight was measured at the end of the intervention with variable durations (MD −2·49 kg, 95% CI −3·34, −1·63) or at 12-months postnatal (MD −2·41 kg, 95% CI −3·89, −0·93) (8). There was also heterogeneity in the time points used to calculate weight change. Pregnancy + Postnatal interventions used pre-pregnancy or early pregnancy weight as the baseline measure, whereas Postnatal Only interventions used weight after delivery. Further, two of the three Pregnancy + Postnatal interventions involved only one intervention session in the postnatal period (18, 22). Minimal intervention in the postnatal period may explain why these studies did not show an effect on postnatal weight management. Interestingly, Huang et al. (26) directly compared a Pregnancy + Postnatal to a Postnatal Only intervention and found that the former produced greater effects on postnatal weight outcomes. A 2018 systematic review identified three studies with Pregnancy + Postnatal interventions and found that one study significantly reduced postnatal weight retention compared to controls at 6-months, but not at 12-months (7). None of the three studies in the 2018 review compared the interventions directly to Postnatal Only interventions. However, results of the study by Huang et al. (26) should be interpreted with caution as we identified several concerns with study quality in the risk of bias assessment (e.g., lack of consideration for confounding variables in the analysis). Also, women in this study had an average pre-pregnancy BMI in the recommended range (approximately BMI 21 kg/m^2^), so we cannot tell if the intervention would be effective in different populations. Huang et al. (26) was one of four studies in this review where women’s mean BMI at study inclusion was in the recommended range (18, 22, 27). One included study had a mean baseline BMI in the overweight range (19), while two had mean baseline BMIs in the obese range (20, 28). Therefore, the findings of our review are applicable to a general postnatal population rather than just higher risk (higher BMI) groups. This is important as weight retention in the postnatal period is a life course driver for longer-term obesity development, and an opportunity to support obesity management. As differences in reporting limited our ability to directly compare intervention effect across different BMI categories, we cannot draw conclusions about the effectiveness of low-intensity interventions across BMI categories. Women with a higher BMI may need more intensive support than women with a lower BMI as we see reflected in the United Kingdom tiered approach to weight management services where intensity of support and the need for specialist health professionals increases with increasing BMI.

Despite methodological heterogeneity, the studies by Berks et al. (19), Daley et al. (20), and Huang et al. (26) showed some promise for weight-related outcomes. These three studies had similar timing, intensity, and duration of intervention delivery; all involved three intervention sessions in the postnatal period that were initiated 2–7 months postnatally and lasted for 2–3 months. While there is no consensus on the optimal timing for postnatal weight management interventions (7), research has shown that four in five women with overweight or obesity plan to seek weight loss information by 4-months postnatal (40). Additionally, returning to pre-pregnancy weight within 6-months postnatal is associated with a lower risk of longer-term obesity (41). A 2018 systematic review found that short/medium duration interventions (i.e., ≤6 months) achieved more weight loss than longer interventions (>12-months) (7). The study that produced the greatest intervention effect was that by Daley et al. (20) (adjusted MD −7.5 kg, 95% CI −13.8 kg to −1.3 kg) where during their child’s routine immunisation visits, women were weighed, provided with brief advice, encouraged to self-monitor weight, and referred to an online weight management programme. Interestingly, the study by Kinnunen et al. (27) which also implemented a counselling intervention in child immunisation clinics (but without self-monitoring or signposting to more intensive support) did not prove effective. It may be that referral to more intensive support and self-monitoring of weight are critical to intervention effectiveness. Indeed, previous work has shown that more intensive interventions help postnatal women to lose a similar amount of weight to Daley et al. (20) and that self-monitoring is as an effective strategy for weight management in this population (39, 42–44). This suggests that using routine primary and community contacts to initiate weight management conversations and signpost to onward support may be an efficient strategy for implementing postnatal weight management support. Previous research has shown that leveraging routine encounters with healthcare professionals is an effective and acceptable way to encourage people to engage with more intensive weight management services. A large United Kingdom based RCT studying an intervention whereby GPs offered patients with obesity a referral to a behavioural weight management programme found that 40% of patients accepted the referral and attended the programme, and patients in the intervention group weighed 1.43 kg (95% CI 0.89–1.97) less than the control group at 12-months follow-up (45). Further, the web-based intervention in Daley et al. (20) that supplemented the brief advice may have enhanced engagement and effectiveness. Digital interventions may overcome some of the barriers faced by postnatal women such as having an unpredictable schedule with a newborn and difficulties leaving the house (20). Use of eHealth technologies by postnatal women has been shown to produce more weight loss than controls (MD −2.55 kg, 95% CI −3.81 to −1.28) after 3 to 12-months (46). As the study by Daley et al. (20) was a feasibility RCT conducted in a small sample (*n* = 28) and the confidence interval for weight loss was wide, caution is advised when interpreting the effect size. An adequately powered trial conducted in a larger sample would increase confidence in the effectiveness and acceptability of this type of intervention.

While intervention adherence was reported by four studies, it is hard to compare adherence across studies and with the wider literature as it was assessed differently in each study. As intervention adherence is one of the most important determinants of successful weight loss and weight loss maintenance (47, 48), reporting of adherence and treatment fidelity needs to be improved in future studies, as a minimum the raw data should be made available so adherence can be estimated. Study attrition rates of the included studies fell within the ranges reported in previous research (49). Interestingly, the studies with markedly lower attrition rates were those by Daley et al. (20) and Kinnunen et al. (27) where interventions were embedded into routine child immunisation visits. This observation is corroborated by previous studies reporting greater retention when interventions were integrated into routine newborn visits (50, 51). Integrating interventions into routine care, including child health care services, may be a particularly effective approach to engage women in postnatal weight management.

### 4.3 Strengths and limitations

The main strength of the current work is the gold standard systematic review methods that were implemented. This includes the involvement of an information specialist for development of the comprehensive search strategy, blinded duplicate screening, data extraction and quality appraisal, and extensive grey literature and citation chaining, and following PRISMA guidelines for reporting. Additionally, we did not restrict our eligibility criteria by study design, which allowed us to gain a holistic overview of evidence relating to the intervention effect, experiences of both participants and workforce, and implementation outcomes. Finally, a multidisciplinary team worked in partnership to provide insight and expertise relating to public health practices, academic topic area expertise in maternal obesity and weight management, and evidence synthesis.

The main limitation of the review was the inability to conduct a meaningful meta-analysis due to the heterogeneity observed between the studies. This was further exacerbated by the limited volume of evidence available and the lack of consistency between the studies regarding the intervention implementation and follow-up. Additionally, data on women’s experience of the intervention was provided by only two studies (19, 20); in the study by Berks et al. (19) these data were collected through a short questionnaire by completers only. The lack of long-term follow-up presents a limitation of the evidence available, with only one study evaluating the effectiveness of their intervention at a time point over 1 year (13 months) (18). Finally, there is no consensus on the definition of intervention intensity. While we based our criteria of ≤1 session/month on a previous review of interventions in primary care settings (11), other reviews have used different definitions for lower-intensity [e.g., <14 sessions over 6 months (52)]. Also, the criteria we applied did not have a threshold for session length. This may have implications on the feasibility of delivering these interventions in real-world health and care settings. More broadly, ambiguity around the definition of intervention intensity limits meaningful comparison across the literature. The development of a standardised framework to describe intervention intensity would help to address this and advance the field of behavioural science and public health science.

### 4.4 Recommendations for further research

One of the main concerns for future research should be to establish longer follow-up periods, as long-term effectiveness data are currently lacking. Such studies should be adequately powered as many of the included studies had small sample sizes. It is especially important that future research presents adjusted analyses for confounding variables as unadjusted analyses are likely to overestimate the impact of the intervention. Future studies should also aim to assess the most appropriate time for starting an intervention and the number of sessions required to produce the most favourable results. In addition, studies should implement standardised weight and behavioural outcomes and measurement tools. The assessment of standardised outcomes and follow-up periods would allow for direct comparisons between studies and any future meta-analyses would benefit from such standardisations.

Four studies reported process outcomes relating to recruitment and adherence (18–20, 28), two for intervention participant experiences (19, 20), and one for staff experiences (20). This information is essential as it provides information regarding what did or did not work, important experiences of participants and staff alike, and aids in the development of future interventions. Therefore, future research should ensure that these experiences are explored and reported alongside intervention effectiveness results using qualitative methods, such as focus groups, to enhance our understanding of the acceptability and implementation of these interventions.

### 4.5 Recommendations for public health and primary care policy/practice

The review highlights that there is limited qualitative evidence on women’s perspective and experience of lower intensity weight management interventions in the period following pregnancy. This gap in understanding could be addressed in part by services involved in both antenatal and postnatal health proactively seeking insights from women as part of their service delivery, for example through developing and utilising Patient Reported Experience Measures (PREMs). In addition, policy makers need to engage with women, families and the public, as well as healthcare professionals, to question, consider, test and revise approaches in this life course period. Interventions initiated within 12-months following a pregnancy, and particularly those at within 6-months postnatal, delivered in routine healthcare appointments, may hold the most promise and warrant further investigation. Understanding the acceptability and feasibility for healthcare professionals to systematically undertake weight management conversations with women after pregnancy would enable policy makers to determine the potential of this approach. Finally, the fragmentation of care between the antenatal and postnatal period, and the potential for weight management interventions to straddle both periods as identified in this review, necessitates that the handover of care needs to be joined up, ensuring support is timely and person-centred.

## Data availability statement

The original contributions presented in the study are included in the article/Supplementary material, further inquiries can be directed to the corresponding author.

## Author contributions

MF: Conceptualization, Data curation, Formal analysis, Investigation, Methodology, Project administration, Writing – original draft, Writing – review & editing. RK: Data curation, Formal analysis, Investigation, Methodology, Project administration, Writing – original draft, Writing – review & editing. KT: Conceptualization, Data curation, Methodology, Writing – review & editing, Validation. AJ: Conceptualization, Formal analysis, Funding acquisition, Writing – review & editing. AL: Conceptualization, Formal analysis, Funding acquisition, Writing – review & editing. MP: Data curation, Writing – review & editing. LS-R: Data curation, Writing – review & editing. GN: Data curation, Writing – review & editing. MA: Data curation, Writing – review & editing. EC: Data curation, Writing – review & editing. HO'K: Data curation, Project administration, Writing – review & editing, Methodology. MM: Data curation, Writing – review & editing. NH: Data curation, Formal analysis, Funding acquisition, Investigation, Methodology, Project administration, Writing – original draft, Writing – review & editing.
