# Supplementary material for: Effectiveness and implementation of lower-intensity weight management interventions delivered by the non-specialist workforce in postnatal women: a mixed-methods systematic review
Source: Front Public Health. 2024 Mar 28;12:1359680. doi: 10.3389/fpubh.2024.1359680 (PMC11008719; doi:10.3389/fpubh.2024.1359680)
Supplement: Supplementary file 1 [file Data_Sheet_1.docx]

Supplementary Material

Effectiveness and implementation of non-specialist interventions delivered in primary and community care settings for postnatal weight management: a systematic review

**Mackenzie Fong^1,2,3^, Ryan Kenny^1,4,5^, Katie Thomson^1,2,3,4,5^, Amrita Jesurasa^6^, Amber Lavans^6^, Maddey Patterson^1,2,3^, Letitia Sermin-Reed^1,2,3^, Gina Nguyen^1,3^, Maria Raisa Jessica Aquino^1,3^, Emer Cullen^1^, Hannah O'Keefe^1,4,5^, Malcolm Moffatt^1,3^, Nicola Heslehurst^1,3*^**

^1^ Population Health Sciences Institute, Faculty of Medical Sciences, Newcastle University, Newcastle-upon-Tyne, United Kingdom

^2^ NIHR Applied Research Collaboration (ARC) North East and North Cumbria, Newcastle-upon-Tyne, United Kingdom

^3^ Fuse, the Centre for Translational Research in Public Health, Newcastle-upon-Tyne, United Kingdom

^4^ NIHR Innovation Observatory, Newcastle University, Newcastle-upon-Tyne, United Kingdom

^5^ Evidence Synthesis Group, Newcastle University, Newcastle-upon-Tyne, United Kingdom

^6^ Primary Care Division, Public Health Wales, Cardiff, United Kingdom

*** Correspondence:**Nicola Heslehurst
nicola.heslehurst@newcastle.ac.uk

# Supplementary File 1: Database search strategies

**Ovid MEDLINE(R) and Epub Ahead of Print, In-Process, In-Data-Review & Other Non-Indexed Citations, Daily and Versions**1946 to Jan, 2023

Via: <https://ovidsp.dc1.ovid.com/ovid-new-a/ovidweb.cgi>

Date range searched: Inception to 9^th^ Jan 2023

Date of search: 9^th^ Jan 2023

Records retrieved: 5994

1 exp Postpartum Period/

2 (postpartum or post-partum or postnatal* or post-natal* or puerperium or postpartal or post-partal or lactating or lactation or "nursing women" or breastfeeding or breast-feeding or "after birth" or "following pregnancy" or postpregnancy or "post pregnancy" or "following childbirth" or "after delivery" or "post childbirth").ti,ab,kw.

3 or/1-2

4 (infant* or pre?school* or "pre?k" or pre?kindergarten or playgroup or "early childhood" or "early years" or "under 5 year*" or neonat* or toddler* or new?born).ti,ab,kw.

5 Child, Preschool/

6 infant/ or infant, newborn/

7 or/3-5

8 mothers/ or adolescent mothers/

9 (mother* or mum* or "birthing person" or maternal*).ti,ab,kw.

10 or/8-9

11 7 and 10

12 3 or 11

13 body-weight trajectory/ or weight gain/ or weight loss/

14 (weight adj2 (manag* or trajectory or loss or lost or losses or lose or loses or fluctuat* or control* or reduc*)).ti,ab,kw.

15 Weight Reduction Programs/

16 or/13-15

17 12 and 16

18 exp Animals/ not Humans/

19 17 not 18

20 limit 19 to english language

**Embase** 1974 to 2023 January 9

Via: <https://ovidsp.dc1.ovid.com/ovid-new-a/ovidweb.cgi>

Date range searched: Inception to 9^th^ Jan 2023

Date of search: 9^th^ Jan 2023

Records retrieved: 6744

1 exp puerperium/

2 (postpartum or post-partum or postnatal* or post-natal* or puerperium or postpartal or post-partal or lactating or lactation or "nursing women" or breastfeeding or breast-feeding or "after birth" or "following pregnancy" or postpregnancy or "post pregnancy" or "following childbirth" or "after delivery" or "post childbirth").ti,ab,kw.

3 or/1-2

4 (infant* or pre?school* or "pre?k" or pre?kindergarten or playgroup or "early childhood" or "early years" or "under 5 year*" or neonat* or toddler* or new?born).ti,ab,kw.

5 preschool child/

6 exp infant/

7 or/3-5

8 mother/ or adolescent mother/

9 (mother* or mum* or "birthing person" or maternal*).ti,ab,kw.

10 or/8-9

11 7 and 10

12 3 or 11

13 body weight change/ or weight gain/ or weight loss/

14 (weight adj2 (manag* or trajectory or loss or lost or losses or lose or loses or fluctuat* or control* or reduc*)).ti,ab,kw.

15 body weight loss/

16 or/13-15

17 12 and 16

18 exp Animals/ not Humans/

19 17 not 18

20 limit 19 to english language

**APA PsycInfo** 1806 to January Week 2 2023

Via: <https://ovidsp.dc1.ovid.com/ovid-new-a/ovidweb.cgi>

Date range searched: Inception to 9^th^ Jan 2023

Date of search: 9^th^ Jan 2023

Records retrieved: 866

1 exp Postnatal Period/

2 (postpartum or post-partum or postnatal* or post-natal* or puerperium or postpartal or post-partal or lactating or lactation or "nursing women" or breastfeeding or breast-feeding or "after birth" or "following pregnancy" or postpregnancy or "post pregnancy" or "following childbirth" or "after delivery" or "post childbirth").ti,ab.

3 or/1-2

4 (infant* or pre?school* or "pre?k" or pre?kindergarten or playgroup or "early childhood" or "early years" or "under 5 year*" or neonat* or toddler* or new?born).ti,ab.

5 Preschool Students/

6 Kindergarten Students/

7 or/3-5

8 mothers/ or adolescent mothers/

9 (mother* or mum* or "birthing person" or maternal*).ti,ab.

10 or/8-9

11 7 and 10

12 3 or 11

13 Body Weight/ or weight gain/ or weight loss/

14 (weight adj2 (manag* or trajectory or loss or lost or losses or lose or loses or fluctuat* or control* or reduc*)).ti,ab.

15 Weight Control/

16 or/13-15

17 12 and 16

18 exp Animals/ not Humans/

19 17 not 18

20 limit 19 to english language

**CINAHL**

Via: <https://search.ebscohost.com/Login.aspx>

Date range searched: Inception to 9^th^ Jan 2023

Date of search: 9^th^ Jan 2023

Records retrieved: 5334

S20 S13 AND S19

S19 S14 OR S15 OR S16 OR S17 OR S18

S18 (MH "Weight Reduction Programs")

S17 TI ( (weight N2 (manag* or trajectory or loss or lost or losses or lose or loses or fluctuat* or control* or reduc*)) ) OR AB ( (weight N2 (manag* or trajectory or loss or lost or losses or lose or loses or fluctuat* or control* or reduc*)) )

S16 (MH "Weight Gain")

S15 (MH "Weight Loss")

S14 (MH "Body Weight")

S13 S3 OR S12

S12 S8 AND S11

S11 S9 OR S10

S10 TI ( (mother* or mum* or "birthing person" or maternal*) ) OR AB ( (mother* or mum* or "birthing person" or maternal*) )

S9 (MH "Mothers") OR (MH "Adolescent Mothers")

S8 S4 OR S5 OR S6 OR S7

S7 (MH "Infant")

S6 (MH "Infant, Newborn")

S5 (MH "Child, Preschool")

S4 TI ( (infant* or pre#school* or "pre#k" or pre#kindergarten or playgroup or "early childhood" or "early years" or "under 5 year*" or neonat* or toddler* or new#born) ) OR AB ( (infant* or pre#school* or "pre#k" or pre#kindergarten or playgroup or "early childhood" or "early years" or "under 5 year*" or neonat* or toddler* or new#born) )

S3 S1 OR S2

S2 TI ( (postpartum or post-partum or postnatal* or post-natal* or puerperium or postpartal or post-partal or lactating or lactation or "nursing women" or breastfeeding or breast-feeding or "after birth" or "following pregnancy" or postpregnancy or "post pregnancy" or "following childbirth" or "after delivery" or "post childbirth") ) OR AB ( (postpartum or post-partum or postnatal* or post-natal* or puerperium or postpartal or post-partal or lactating or lactation or "nursing women" or breastfeeding or breast-feeding or "after birth" or "following pregnancy" or postpregnancy or "post pregnancy" or "following childbirth" or "after delivery" or "post childbirth") )

S1 (MH "Postnatal Period+")

**Cochrane Library** (CENTRAL, CDSR)

Via: <https://www.cochranelibrary.com/advanced-search>

Date range searched: Inception to 9^th^ Jan 2023

Date of search: 9^th^ Jan 2023

Records retrieved: 38 publications, 1627 trials

ID Search Hits

#1 MeSH descriptor: [Postpartum Period] explode all trees 1942

#2 ((postpartum or post-partum or postnatal* or post-natal* or puerperium or postpartal or post-partal or lactating or lactation or "nursing women" or breastfeeding or breast-feeding or "after birth" or "following pregnancy" or postpregnancy or "post pregnancy" or "following childbirth" or "after delivery" or "post childbirth")):ti,ab,kw 32183

#3 {OR #1-#2} 32184

#4 ((infant* or pre?school* or "pre?k" or pre?kindergarten or playgroup or "early childhood" or "early years" or "under 5 year*" or neonat* or toddler* or new?born)):ti,ab,kw 108562

#5 MeSH descriptor: [Child, Preschool] this term only 31658

#6 MeSH descriptor: [Infant, Newborn] this term only 17631

#7 MeSH descriptor: [Infant] this term only 23883

#8 {OR #4-#7} 108562

#9 MeSH descriptor: [Mothers] this term only 2259

#10 MeSH descriptor: [Adolescent Mothers] this term only 5

#11 ((mother* or mum* or "birthing person" or maternal*)):ti,ab,kw 43123

#12 ^10-#11^ 43123

#13 #8 AND #12 23268

#14 #3 OR #13 46211

#15 MeSH descriptor: [Body-Weight Trajectory] this term only 17

#16 MeSH descriptor: [Weight Loss] this term only 6947

#17 MeSH descriptor: [Weight Gain] this term only 2775

#18 ((weight NEAR/2 (manag* or trajectory or loss or lost or losses or lose or loses or fluctuat* or control* or reduc*))):ti,ab,kw 33183

#19 MeSH descriptor: [Weight Reduction Programs] this term only 885

#20 {OR #15-#19} 35125

#21 #14 AND #20 1659

**Scopus**

Via: [https://www-scopus-com.](https://www-scopus-com.libproxy.ncl.ac.uk/search/form.uri?display=basic#basic)

Date range searched: Inception to 9^th^ Jan 2023

Date of search: 9^th^ Jan 2023

Records retrieved: 2240

( ( TITLE-ABS-KEY ( postpartum OR post-partum OR postnatal* OR post-natal* OR puerperium OR postpartal OR post-partal OR lactating OR lactation OR "nursing women" OR breastfeeding OR breast-feeding OR "after birth" OR "following pregnancy" OR postpregnancy OR "post pregnancy" OR "following childbirth" OR "after delivery" OR "post childbirth" ) ) OR ( ( TITLE-ABS-KEY ( infant* OR pre?school* OR "pre?k" OR pre?kindergarten OR playgroup OR "early childhood" OR "early years" OR "under 5 year*" OR neonat* OR toddler* OR new?born ) ) AND ( TITLE-ABS-KEY ( mother* OR mum* OR "birthing person" OR maternal* ) ) ) ) AND ( TITLE-ABS-KEY ( weight W/2 ( manag* OR trajectory OR loss OR lost OR losses OR lose OR loses OR fluctuat* OR control* OR reduc* ) ) ) AND NOT INDEX ( medline ) AND ( LIMIT-TO ( LANGUAGE , "English" ) )

**ProQuest** Dissertation & Thesis

Via: https://www.proquest.com/

Date range searched: Inception to 9^th^ Jan 2023

Date of search: 9^th^ Jan 2023

Records retrieved: 37

((title((postpartum OR post-partum OR postnatal* OR post-natal* OR puerperium OR postpartal OR post-partal OR lactating OR lactation OR "nursing women" OR breastfeeding OR breast-feeding OR "after birth" OR "following pregnancy" OR postpregnancy OR "post pregnancy" OR "following childbirth" OR "after delivery" OR "post childbirth")) OR abstract((postpartum OR post-partum OR postnatal* OR post-natal* OR puerperium OR postpartal OR post-partal OR lactating OR lactation OR "nursing women" OR breastfeeding OR breast-feeding OR "after birth" OR "following pregnancy" OR postpregnancy OR "post pregnancy" OR "following childbirth" OR "after delivery" OR "post childbirth"))) OR ((title((infant* OR pre?school* OR "pre?k" OR pre?kindergarten OR playgroup OR "early childhood" OR "early years" OR "under 5 year*" OR neonat* OR toddler* OR new?born)) OR abstract((infant* OR pre?school* OR "pre?k" OR pre?kindergarten OR playgroup OR "early childhood" OR "early years" OR "under 5 year*" OR neonat* OR toddler* OR new?born))) AND (title((mother* OR mum* OR "birthing person" OR maternal*)) OR abstract((mother* OR mum* OR "birthing person" OR maternal*))))) AND (title((weight NEAR/2 (manag* OR trajectory OR loss OR lost OR losses OR lose OR loses OR fluctuat* OR control* OR reduc*))) OR abstract((weight NEAR/2 (manag* OR trajectory OR loss OR lost OR losses OR lose OR loses OR fluctuat* OR control* OR reduc*))))

**MIDIRS**

Via: <https://www.midirs.org/informing/midirs-midwifery-digest/>

Date range searched: Inception to 9^th^ Jan 2023

Date of search: 9^th^ Jan 2023

Records retrieved: 6

Postpartum weight management

Weight management

Weight loss

Weight

**ClinicalTrials.gov**

Via: <https://clinicaltrials.gov/>

Date range searched: Inception to 9^th^ Jan 2023

Date of search: 9^th^ Jan 2023

Records retrieved: 157

Postpartum weight management

Puerperium weight management

# Supplementary File 2: Grey Literature Sources

Grey literature sources were searched using two terms: Postpartum weight; Puerperium weight.

**Office for Health Improvement and Disparities**

Via: <https://www.gov.uk/government/organisations/office-for-health-improvement-and-disparities>

Date range searched: Inception to 25^th^ Jan 2023

Date of search: 25^th^ Jan 2023

Records retrieved: 0

**Public Health Wales**

Via: <https://phw.nhs.wales/>

Date range searched: Inception to 25^th^ Jan 2023

Date of search: 25^th^ Jan 2023

Records retrieved: 172

**Public Health Scotland**

Via: <https://publichealthscotland.scot/>

Date range searched: Inception to 25^th^ Jan 2023

Date of search: 25^th^ Jan 2023

Records retrieved: 0

**Public Health Northern Ireland**

Via: <https://www.publichealth.hscni.net/>

Date range searched: Inception to 25^th^ Jan 2023

Date of search: 25^th^ Jan 2023

Records retrieved: 0

**Public Health Republic of Ireland**

Via: <https://www.hse.ie/>

Date range searched: Inception to 25^th^ Jan 2023

Date of search: 25^th^ Jan 2023

Records retrieved: 3044

**World Health Organisation**

Via: <https://www.who.int/>

Date range searched: Inception to 25^th^ Jan 2023

Date of search: 25^th^ Jan 2023

Records retrieved: 0

**UNICEF**

Via: <https://www.unicef.org/evaluation/reports#/>

Date range searched: Inception to 25^th^ Jan 2023

Date of search: 25^th^ Jan 2023

Records retrieved: 0

**National Institute of Health and Care Excellence**

Via: <https://www.nice.org.uk/>

Date range searched: Inception to 25^th^ Jan 2023

Date of search: 25^th^ Jan 2023

Records retrieved: 0

**NHS England Integrated care systems: case studies**

Via: <https://www.england.nhs.uk/integratedcare/resources/case-studies/>

Date range searched: Inception to 25^th^ Jan 2023

Date of search: 25^th^ Jan 2023

Records retrieved: 550

**The collective for action on obesity: Obesity Australia**

Via: <https://www.worldobesity.org/our-network/our-members/the-collective-for-action-on-obesity-obesity-australia/>

Date range searched: Inception to 26th Jan 2023

Date of search: 26th Jan 2023

Records Retrieved: 0

**Belgium association for the study of obesity BASO**

Via: <https://www.worldobesity.org/our-network/our-members/belgium-association-for-the-study-of-obesity-baso/>

Date range searched: Inception to 26th Jan 2023

Date of search: 26th Jan 2023

Records Retrieved: 0

**Hong Kong association for the study of obesity HKASO**

Via: <https://www.worldobesity.org/our-network/our-members/hong-kong-association-for-the-study-of-obesity-hkaso/>

Date range searched: Inception to 26th Jan 2023

Date of search: 26th Jan 2023

Records Retrieved: 0

**Hongkong obesity society**

Via: <https://www.worldobesity.org/our-network/our-members/hong-kong-obesity-society/>

Date range searched: Inception to 26th Jan 2023

Date of search: 26th Jan 2023

Records Retrieved: 0

**Japan society for the study of obesity**

Via: <https://www.worldobesity.org/our-network/our-members/japan-society-for-the-study-of-obesity/>

Date range searched: Inception to 26th Jan 2023

Date of search: 26th Jan 2023

Records Retrieved: 0

**Dasman diabetes institute Kuwait**

Via: <https://www.worldobesity.org/our-network/our-members/dasman-diabetes-institute-kuwait/>

Date range searched: Inception to 26th Jan 2023

Date of search: 26th Jan 2023

Records Retrieved: 0

**Netherlands association for the study of obesity naso**

Via: <https://www.worldobesity.org/our-network/our-members/netherlands-association-for-the-study-of-obesity-naso/>

Date range searched: Inception to 26th Jan 2023

Date of search: 26th Jan 2023

Records Retrieved: 0

**World Obesity member: Australia**

Via: <https://www.worldobesity.org/our-network/our-members/australia/>

Date range searched: Inception to 26th Jan 2023

Date of search: 26th Jan 2023

Records Retrieved: 0

**Asia Oceania association for the study of obesity AOASO**

Via: <https://www.worldobesity.org/our-network/our-members/asia-oceania-association-for-the-study-of-obesity-aoaso/>

Date range searched: Inception to 26th Jan 2023

Date of search: 26th Jan 2023

Records Retrieved: 0

**Gasol foundation**

Via: <https://www.worldobesity.org/our-network/our-members/gasol-foundation/>

Date range searched: Inception to 26th Jan 2023

Date of search: 26th Jan 2023

Records Retrieved: 0

**The international federation for the surgery of obesity and metabolic disorders IFSO**

Via: <https://www.worldobesity.org/our-network/our-members/the-international-federation-for-the-surgery-of-obesity-and-metabolic-disorders-ifso/>

Date range searched: Inception to 26th Jan 2023

Date of search: 26th Jan 2023

Records Retrieved: 0

**Global obesity patient alliance GOPA**

Via: <https://www.worldobesity.org/our-network/our-members/global-obesity-patient-alliance-gopa/>

Date range searched: Inception to 26th Jan 2023

Date of search: 26th Jan 2023

Records Retrieved: 0

**Healthy Caribbean coalition HCC**

Via: <https://www.worldobesity.org/our-network/our-members/healthy-caribbean-coalition-hcc/>

Date range searched: Inception to 26th Jan 2023

Date of search: 26th Jan 2023

Records Retrieved: 1

**Association for the study of obesity ASO**

Via: <https://www.worldobesity.org/our-network/our-members/association-for-the-study-of-obesity-aso/>

Date range searched: Inception to 26th Jan 2023

Date of search: 26th Jan 2023

Records Retrieved: 2

**The obesity society TOS USA**

Via: <https://www.worldobesity.org/our-network/our-members/the-obesity-society-tos-usa/>

Date range searched: Inception to 26th Jan 2023

Date of search: 26th Jan 2023

Records Retrieved: 1

**Singapore association for the study of obesity SASO**

Via: <https://www.worldobesity.org/our-network/our-members/singapore-association-for-the-study-of-obesity-saso/>

Date range searched: Inception to 26th Jan 2023

Date of search: 26th Jan 2023

Records Retrieved: 2

**Korean society for the study of obesity KSSO**

Via: <https://www.worldobesity.org/our-network/our-members/korean-society-for-the-study-of-obesity-ksso/>

Date range searched: Inception to 26th Jan 2023

Date of search: 26th Jan 2023

Records Retrieved: 3

**European association for the study of obesity EASO**

Via: <https://www.worldobesity.org/our-network/our-members/european-association-for-the-study-of-obesity-easo/>

Date range searched: Inception to 26th Jan 2023

Date of search: 26th Jan 2023

Records Retrieved: 3

**Obesity action coalition**

Via: <https://www.worldobesity.org/our-network/our-members/obesity-action-coalition/>

Date range searched: Inception to 26th Jan 2023

Date of search: 26th Jan 2023

Records Retrieved: 6

**Obesity Canada**

Via: <https://www.worldobesity.org/our-network/our-members/obesity-canada/>

Date range searched: Inception to 26th Jan 2023

Date of search: 26th Jan 2023

Records Retrieved: 11

**The association for the study of obesity in Ireland**

Via: <https://www.worldobesity.org/our-network/our-members/the-association-for-the-study-of-obesity-in-ireland/>

Date range searched: Inception to 26th Jan 2023

Date of search: 26th Jan 2023

Records Retrieved: 20

**Malta nutrition and obesity research unit**

Via: <https://www.worldobesity.org/our-network/our-members/malta-nutrition-and-obesity-research-unit/>

Date range searched: Inception to 26th Jan 2023

Date of search: 26th Jan 2023

Records Retrieved: 85

**Royal College of Nursing**

Via: <https://www.rcn.org.uk/>

Date range searched: Inception to 27^th^ Jan 2023

Date of search: 27^th^ Jan 2023

Records retrieved: 0

**Royal College of Primary Care**

Via: <https://www.rcgp.org.uk/>

Date range searched: Inception to 27^th^ Jan 2023

Date of search: 27^th^ Jan 2023

Records retrieved: 0

**Institute of Health Visiting**

Via: <https://ihv.org.uk/>

Date range searched: Inception to 27^th^ Jan 2023

Date of search: 27^th^ Jan 2023

Records retrieved: 70

**Wellbeing of Women**

Via: <https://www.wellbeingofwomen.org.uk/>

Date range searched: Inception to 27^th^ Jan 2023

Date of search: 27^th^ Jan 2023

Records retrieved: 0

**World Obesity Federation**

Via: <https://www.worldobesity.org/>

Date range searched: Inception to 30^th^ Jan 2023

Date of search: 30^th^ Jan 2023

Records retrieved: 32

**Obesity Health Alliance**

Via: <https://obesityhealthalliance.org.uk/>

Date range searched: Inception to 30^th^ Jan 2023

Date of search: 30^th^ Jan 2023

Records retrieved: 0

**Google searches for local gov.uk and ac.uk sites (incognito mode)**

Date range searched: Inception to 10^th^ Feb 2023

Date of search: 10^th^ Feb 2023

Records retrieved: first 100 for each search (400 total)

postpartum weight + site:gov.uk

puerperium weight + site:gov.uk

postpartum weight + thesis OR dissertation OR report + site:ac.uk

puerperium weight + thesis OR dissertation OR report + site:ac.uk

# 3. Supplementary Tables

**Supplementary Table 1.** **Characteristics of interventions of included studies**

| **Author (year) country** | **Intervention description and format** | **Intervention setting** | **Intervention deliverer** | **Intervention delivered in routine care?** | **Study follow-up duration (and/or postnatal time point)** |
| --- | --- | --- | --- | --- | --- |
| Althuizen *et al.* (2013)(1)  Netherlands | The New Life(style) programme consisted of 5 individual counselling modules. These discussed how to control weight gain during and after pregnancy, and how to maintain or optimise a healthy lifestyle. Attended face-to-face appointments at 18, 22, 30, and 36 weeks of gestation, telephone session at 8 weeks postnatal. Each session was approx. 15 minutes. First session was approx. 30 mins. Provided with brochure in first session summarising it. | Midwifery practice (during pregnancy)  Home (telephone call postnatally) | Counsellors who were part of the research team | No | 18-month follow-up (12 months postnatal) |
| Berks *et al.* (2019)(2)  Netherlands | 3 sessions, 7, 8.5 and 10 months postnatal. Counselling sessions based on motivational interviewing and the use of a computer-tailored Dutch health education program. Computer program and health questionnaires completed before each counselling session. Information used to set personal lifestyle goals. Progress was discussed and if applicable adjusted, at each subsequent session. At 10-month visit the screening for cardiometabolic risk factors was repeated with exclusion of the lifestyle questionnaire. | Secondary of tertiary hospital, or telephone if necessary | Trained lifestyle counsellor | No | 7-months follow-up (13 months postnatal) |
| Daley *et al.* (2021)(3); Daley *et al.* (2020) (4)  United Kingdom | Brief support to encourage active self-management of their weight at child immunisation appointments (2, 3, 4 and 12 months). During the first three appointments nurses encouraged participants to make healthier lifestyle choices and signposted them to a validated weight management programme (Positive Online Weight Reduction, POWeR) for support. Participants were asked to weigh themselves weekly and record the weight in the child’s health record or in the online tool. Intervention took place until the 4-month immunisation visit. | GP Practice: child immunisation clinics | Practice nurses | Yes | 3-month follow-up (approx. 4.5 months postnatal) |
| Hoffman *et al.* (2019)(5); Hoffman *et al.* (2020)(6); Kunath *et al.* (2019)(7); Rauh *et al.* (2014)(8)  Germany | Alongside routine care visits women received three antenatal (12-16th, 16-20th and 30-34th week of gestation) and one postnatal (6-8 weeks postnatal) face-to-face counselling sessions lasting between 30 and 45 minutes. Women were informed about adequate GWG, breastfeeding, healthy diet and physical activity behaviour during pregnancy and postnatally. Women received a weight gain chart for self-monitoring of weight as proposed by the IOM. At the postnatal visit, women received information on nutrition for breastfeeding. Weight was measured and recorded at all visits. | Gynaecological and midwifery practices | Trained midwives, medical personnel, or gynaecologists | Yes | 18-months follow-up (12 months postnatal) |
| Huang *et al.* (2011)(9)  Taiwan | Intervention 1: nurse designed individualised dietary and physical activity plan based on the baseline information. Six one-to-one counselling sessions (16 weeks, 28 weeks, 36-38 weeks gestation, before hospital discharge, six weeks postnatal, and 3 months postnatal). Each session was about 30-40 minutes. At each booster session participants submitted three-day records of their diet and self-monitored activity.  Intervention 2: Same intervention but began 24-48 hours after birth and extended to 6 months pp. Conducted at the bedside and during regularly scheduled clinic visits. Involved one primary counselling session, one brochure and two booster sessions at 6 weeks pp and 3 months pp | Hospital: obstetric clinics | Masters educated nurse with training in nutrition and physical fitness | Yes | Intervention 1: 12-month follow-up (6-months postnatal)  Intervention 2: 6-month follow-up (6 months postnatal) |
| Kinnunen *et al.* (2007)(10)  Finland | Brief discussions about pre-pregnancy body weight at the child’s 2-month visit to the clinic. If pre-pregnancy weight was lower than current weight the PHN encouraged the participant to try to return to that weight with the help of dietary and physical activity objectives. Physical activity and dietary counselling was 1 primary session (20-30mins) at the 2-month immunisation visit and four booster sessions (10-15 mins) at 3, 5, 6, and 10 month visits. | Child health clinic | Public health nurse | Yes | 8-months follow-up (10 months postnatal) |
| Lohr *et al.* (2021)(11)  United States | Participants received a tablet device, blood pressure equipment, and a Bluetooth-enabled scale and were instructed to take daily blood pressure and weight measurements which were transmitted to a central monitoring site daily and were reviewed by trained nurses; parent study did not consider this as an intervention; however, daily weight were obtained using the Bluetooth-scale. | Home-based | Self-delivered | No | 6-weeks follow-up (6-weeks postnatal) |

**Footnote:** Abbreviations: PHN = public health nurse

**Supplementary Table 2. List of outcomes reported by included studies**

| **Author (year) country** | **Weight-Related Outcomes** | **Diet/physical activity outcomes** | **Intervention implementation outcomes** | **Experience of intervention** |
| --- | --- | --- | --- | --- |
| Althuizen *et al.* (2013)(1)  Netherlands | - Weight - BMI - % women retaining > 3kg of pre-pregnancy weight | NR | - Intervention adherence | NR |
| Berks *et al.* (2019)(2)  Netherlands | - Weight - Waist-to-hip ratio | NR | - % eligible women who completed the intervention - Participation rate - Intervention adherence | - Women’s experience of receiving the intervention |
| Daley *et al.* (2021)(3); Daley *et al.* (2020)(4)  United Kingdom | - Weight - Body fat | - Three Factor Eating Questionnaire - Pregnancy Physical Activity Questionnaire - Weight Control Strategies Scale | - Recruitment rate - Adherence to weekly self-weighing - Adherence to registration with POWeR - Completion rate | - Women’s experience of receiving the intervention - Nurses and GP experience of receiving intervention training and delivering intervention |
| Hoffman *et al.* (2019)(5); Hoffman *et al.* (2020)(6); Kunath *et al.* (2019)(7); Rauh *et al.* (2014)(8)  Germany | - PNWR | NR | NR | NR |
| Huang *et al.* (2011)(9)  Taiwan | - Weight - PNWR | - Health promoting behaviour (nutrition and physical activity) - Self-efficacy (nutrition and physical activity) | NR | NR |
| Kinnunen *et al.* (2007)(10)  Finland | - % women returning to pre-pregnancy weight - PNWR - Waist circumference | - Dietary intake - Leisure time physical activity | NR | NR |
| Lohr *et al.* (2021)(11)  United States | - Weight - BMI | NR | - % women self-weighing - Compliance with daily weighing | NR |

**Footnote:** Abbreviations: GWG = gestational weight gain; NR = not reported; PNWR = postnatal weight retention

**Supplementary Table 3. Intervention and study attrition rates.**

| **Author (year) country** | **Intervention** | **Drop-out at end of intervention** | **Drop-out at last follow-up (intervention)** | **Drop-out at last follow-up (control)** | **Drop-out at last follow-up (total)** |
| --- | --- | --- | --- | --- | --- |
| Althuizen *et al.* (2013)(1)  Netherlands | 4x face-to-face antenatal counselling sessions.  1x PN telephone call. | NR | 21.1% (n = 26) | 18.7% (n = 23) | 19.9% (n = 49) |
| Berks *et al.* (2019)(2)  Netherlands | 3x PN counselling sessions (preferably face-to-face), supported by a computer-tailored health programme and questionnaires. | 28% (n = 40) | 34.7% (n = 50) | 48.4% (n = 30) | 38.8% (n = 80) |
| Daley *et al.* (2021); (3) Daley *et al.* (2020)(4)  United Kingdom | 3 x face-to-face PN brief counselling at child immunisation appointment with signposting to POWeR online tool and self-weigh weekly. | 6.3% (n = 1) | 6.3% (n = 1) | 0 | 3.6% (n = 1) |
| Hoffman *et al.* (2019)(5); Hoffman *et al.* (2020)(6); Kunath *et al.* (2019)(7); Rauh et al (2014)(8)  Germany | 3x face-to-face antenatal counselling sessions.  1x face-to-face PN counselling session. | 11.9% (n = 136) | 20.8% (237) | 21.5% (n = 241) | 21.1% (n = 478) |
| Huang *et al.* (2011)(9)  Taiwan | Intervention 1:  3x face-to-face antenatal counselling sessions.  3x face-to-face PN counselling sessions.  Intervention 2:  3x face-to-face PN counselling sessions, plus a brochure. | Intervention 1: 23.8% (n = 19)  Intervention 2: 20.0% (n = 16) | Intervention 1: 23.8% (n = 19)  Intervention 2: 20.0% (n = 16) | 20.0% (n = 16) | 21.3% (n = 51) |
| Kinnunen *et al.* (2007)(10)  Finland | 4x face-to-face PN counselling sessions at child clinical visits. | 9.4% (n = 5) | 9.4% (n = 5) | 5.1% (n = 2) | 7.6% (n = 7) |
| Lohr *et al.* (2021)(11)  United States | Instructed to self-weigh daily. | NR | NR | NR | NR |

**Footnote:** Abbreviations: NR = not reported; PN = postnatal

**Supplementary Table 4. Intervention implementation and process outcomes**

| **Author (year)**  **country** | **Intervention** | **Outcome** | **Intervention** | **Control** |
| --- | --- | --- | --- | --- |
| Althuizen *et al.* (2013)(1)  Netherlands | 4x face-to-face antenatal counselling sessions.  1x PN telephone call. | Intervention adherence^*^ | 6% (n = 7) attended 0-1 session  8% (n = 10) attended 2-3 sessions  19% (n = 23) attended 4 sessions  67% (n = 83) attended all sessions | NA |
| Berks *et al.* (2019)(2)  Netherlands | 3x PN counselling sessions (preferably face-to-face), supported by a computer-tailored health programme and questionnaires. | Intervention completion^$^ | 23% (n = 94) | NA |
|  |  | Adherence† | 65% (n = 94) | 52% (n = 32) |
|  |  | Participation rate^¶^ | 35% (n = 144) | NA |
| Daley *et al.* (2021)(3); Daley *et al.* (2020)(4)  United Kingdom | 3x face-to-face PN brief counselling at child immunisation appointment with signposting to POWeR online tool and self-weigh weekly. | Participant recruitment rate^‡^ | 35% (95% CI = 25-45%) (n = 28) for the total sample | |
|  |  | Participant intervention adherence: weekly self-weighing^§^ | Self-weighing: 63% (95% CI = 39-86%) (n = 10) | NA |
|  |  | Participant intervention adherence: registration with POWeR | 56% (95% CI = 32-81%) (n = 9) | NA |
|  |  | Intervention delivery adherence: nurse weighed women and recorded weight^#^ | 69% (n = 11^**^) | NA |
|  |  | Intervention delivery adherence: nurse checked participant self-weighing | 88.2% (n = 15^**^) | NA |
|  |  | Intervention delivery adherence: nurse asked women if they accessed POWeR | 88.2% (n = 15^**^) | NA |
|  |  | Intervention delivery adherence: nurse signposted participant to POWeR | 88.2% (n = 15^**^) | NA |
| Lohr *et al.* (2021)(11)  United States | Instructed to self-weigh daily. | Participant intervention adherence: self-weighing ^$$^ | 77% (n = 165) | NA |
|  |  | Participant intervention adherence: frequency of self-weighing | 4.8 times per week | NA |

**Footnote:**^*^of women in the intervention group (n = 123). ^$^proportion of women who completed the intervention out of total eligible women (n = 407). †proportion of women who attended 13-month visit out of women who agreed to participate (n = 144 intervention; n = 62 control). ^¶^proportion of women who agreed to participate in the study out of total women who were eligible for participation (n = 407). ^‡^proportion of participants who took part in the trial out of the recruitment target (n = 80). ^§^self-weighed weekly ≥ 60% of the time. ^#^ nurse weighed women and recorded weight ≥ 60% of the time. ^**^out of total number of recorded consultations (n = 17). ^$$^self-weighed ≥ 4 times/week. Abbreviations: PN = postnatal; NA = not applicable.

# Supplementary Figures

## Supplementary Figure 1. Risk of Bias (RoB) assessment for RCTs


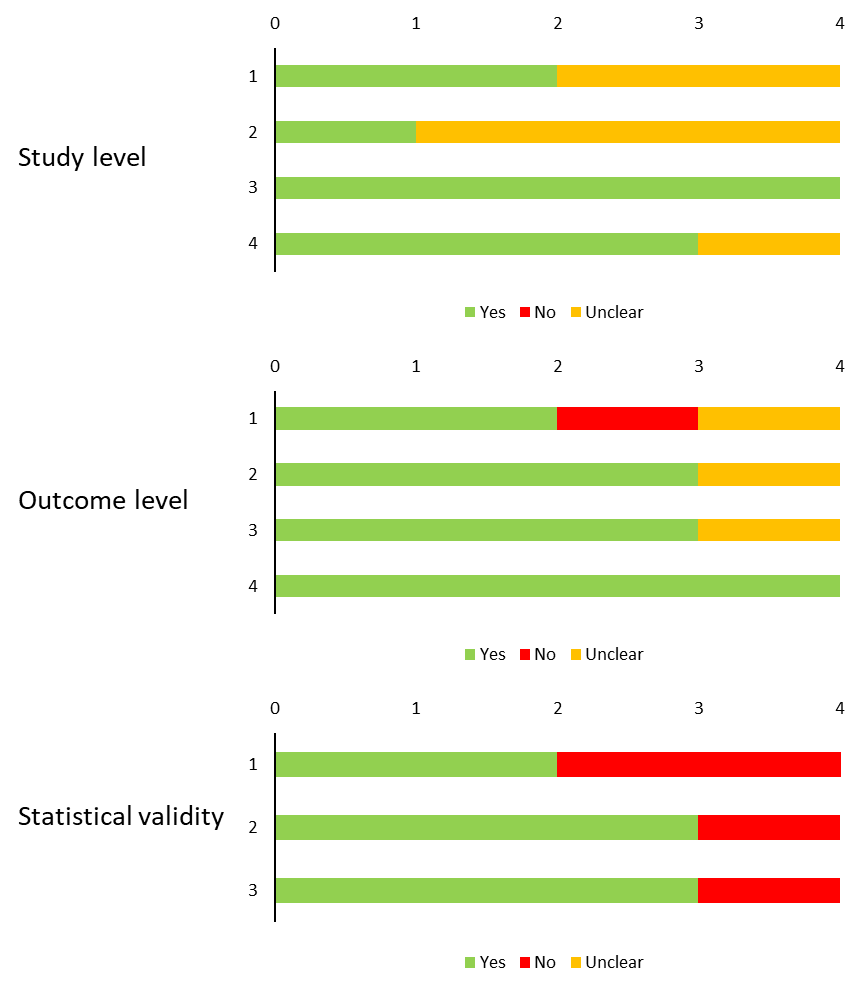


**Supplementary Figure 1.** Overview of risk of bias assessment for RCTs; x-axis represents number of studies; y-axis corresponds to the following questions: A. Study level: 1. Was true randomisation used for assignment of participants to treatment groups? 2. Was allocation to treatment groups concealed? 3. Were treatment groups similar at the baseline? 4. Were treatment groups treated identically other than the intervention of interest? B. Outcome level: 1. Were outcome assessors blind to treatment assignment? 2. Were outcomes measured in the same way for treatment groups?

3. Were outcomes measured in a reliable way? 4. Was follow up complete and if not, were differences between groups in terms of their follow up adequately described and analysed? C. Statistical validity: 1. Were participants analysed in the groups to which they were randomized? 2. Was appropriate statistical analysis used? 3. Was the trial design appropriate and any deviations from the standard RCT design (individual randomization, parallel groups) accounted for in the conduct and analysis of the trial?

**
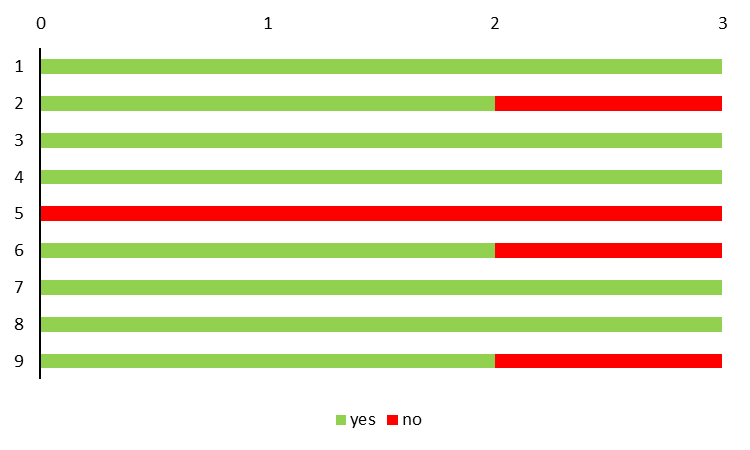
Supplementary Figure 2. Risk of Bias (RoB) assessment for quasi-experimental studies**

**Supplementary Figure 2.** Overview of risk of bias assessment for quasi-experimental studies, y-axis corresponds to the following questions below: 1. Is it clear in the study what is the ‘cause’ and what is the ‘effect’ (i.e. there is no confusion about which variable comes first)? 2. Were the participants included in any comparisons similar? 3. Were the participants included in any comparisons receiving similar treatment/care, other than the exposure or intervention of interest? 4. Was there a control group? 5. Were there multiple measurements of the outcome both pre and post the intervention/exposure? 6. Was follow up complete and if not, were differences between groups in terms of their follow up adequately described and analysed? 7. Were the outcomes of participants included in any comparisons measured in the same way? 8. Were outcomes measured in a reliable way? 9. Was appropriate statistical analysis used?

**References**

1. Althuizen E, van der Wijden CL, van Mechelen W, Seidell JC, van Poppel MNM. The effect of a counselling intervention on weight changes during and after pregnancy: a randomised trial. BJOG : an international journal of obstetrics and gynaecology. 2013;120(1):92-9.

2. Berks D, Hoedjes M, Raat H, Franx A, Looman CWN, Van Oostwaard MF, et al. Feasibility and effectiveness of a lifestyle intervention after complicated pregnancies to improve risk factors for future cardiometabolic disease. Pregnancy Hypertension. 2019;15:98-107.

3. Daley AJ, Jolly K, Ives N, Jebb SA, Tearne S, Greenfield SM, et al. Practice nurse-supported weight self-management delivered within the national child immunisation programme for postnatal women: a feasibility cluster RCT. Health technology assessment (Winchester, England). 2021;25(49):1-130.

4. Daley AJ, Jolly K, Bensoussane H, Ives N, Jebb SA, Tearne S, et al. Feasibility and acceptability of a brief routine weight management intervention for postnatal women embedded within the national child immunisation programme in primary care: randomised controlled cluster feasibility trial. Trials. 2020;21(1):757.

5. Hoffmann J, Gunther J, Stecher L, Spies M, Meyer D, Kunath J, et al. Effects of a Lifestyle Intervention in Routine Care on Short- and Long-Term Maternal Weight Retention and Breastfeeding Behavior-12 Months Follow-up of the Cluster-Randomized GeliS Trial. Journal of clinical medicine. 2019;8(6).

6. Hoffmann J, Gunther J, Stecher L, Spies M, Meyer D, Raab R, et al. Does an antenatal lifestyle intervention in routine care improve maternal and infant health outcomes in the first year postpartum-12 months follow-up of the cluster-randomised GeliS trial. Obesity Reviews. 2020;21.

7. Kunath J, Günther J, Rauh K, Hoffmann J, Stecher L, Rosenfeld E, et al. Effects of a lifestyle intervention during pregnancy to prevent excessive gestational weight gain in routine care - the cluster-randomised GeliS trial. BMC medicine. 2019;17(1):5.

8. Rauh K, Kunath J, Rosenfeld E, Kick L, Ulm K, Hauner H. Healthy living in pregnancy: a cluster-randomized controlled trial to prevent excessive gestational weight gain - rationale and design of the GeliS study. BMC pregnancy and childbirth. 2014;14:119.

9. Huang T-t, Yeh C-Y, Tsai Y-C. A diet and physical activity intervention for preventing weight retention among Taiwanese childbearing women: a randomised controlled trial. Midwifery. 2011;27(2):257-64.

10. Kinnunen TI, Pasanen M, Aittasalo M, Fogelholm M, Weiderpass E, Luoto R. Reducing postpartum weight retention--a pilot trial in primary health care. Nutrition journal. 2007;6:21.

11. Lohr AN, Hoppe KK, Mei CC, Antony KM. Does Daily Self-Weighing Contribute to Postpartum Weight Loss? A Secondary Analysis of Daily Postpartum Weights among Women with Hypertensive Disorders of Pregnancy. American journal of perinatology. 2021.
